# On astrocyte-neuron interactions: Broad insights from the striatum

**DOI:** 10.1016/j.neuron.2025.08.009

**Published:** 2025-09-18

**Authors:** Baljit S. Khakh

**Affiliations:** 1 Department of Physiology and Department of Neurobiology, David Geffen School of Medicine, University of California Los Angeles, Los Angeles, CA, USA

## Abstract

A long-standing question in biology and medicine concerns how astrocytes influence neurons. Here, progress concerning how astrocytes affect neurons and neural circuits is summarized by focusing on data and concepts from studies of the striatum, which has emerged as a model nucleus. Mechanisms broadly applicable across brain regions and disorders are emphasized, and knowledge gaps are described. Experiments spanning multiple scales of biology show that astrocytes regulate neural circuits by virtue of homeostatic signaling and through astrocyte-neuron interactions. During disease, astrocytes contribute to nervous system malfunction in context-specific ways through failures of normal functions and the development of maladaptive responses. As ideally positioned endogenous cellular neuromodulators, astrocytes can be targeted for strategies to regulate neural circuits in brain disorders. After a historically slow start for the field, astrocyte-neuron interactions are now recognized as consequential for physiology and behavior, critically involved in pathophysiology, and exploitable in disease.

## INTRODUCTION

**Figure 1.**
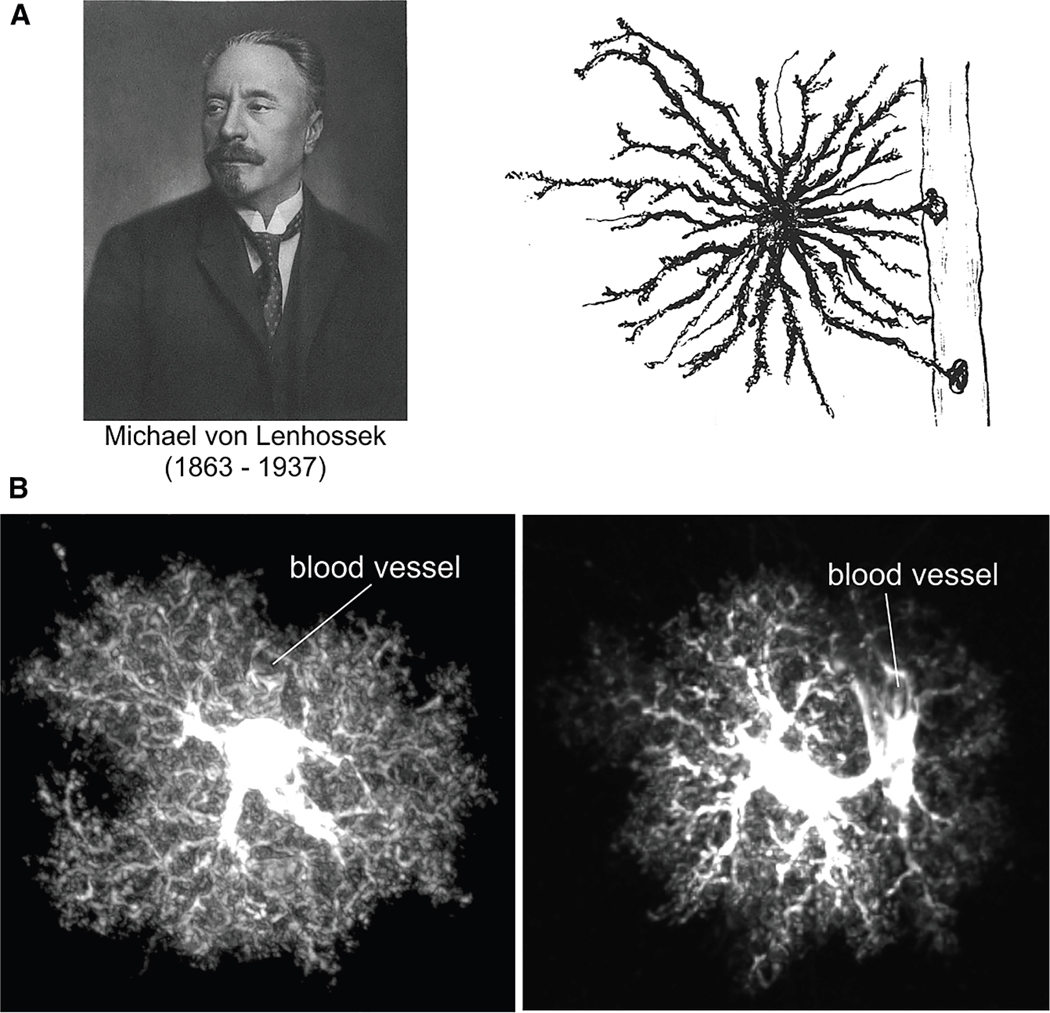
Michael von Lenhossek and early drawings of astrocytes, along with more recent images (A) Photograph of Michael von Lenhossek, who named astrocytes. The right panel shows one of Lenhossek’s drawings of a protoplasmic astrocyte touching a blood vessel (stained with the Golgi technique). (B) Images of striatal astrocytes impaled with sharp glass electrodes, loaded with lucifer yellow iontophoresis and imaged with confocal microscopy. (A) is adapted from Verkhratsky and Butt. ^2^ (B) is from Chai et al. ^8^ and Soto et al. ^9^

Astrocytes, identified by European neuroanatomists at the dawn of neuroscience, ^1,2^ are the largest population of a group of cells within the central nervous system (CNS) known as glia, which also includes significant numbers of oligodendrocytes and microglia. ^3^ Glia were documented by several illustrious neuroscientists in the late 1800s and early 1900s, and astrocytes were named as a distinct cell type by Michael von Lenhossek ^2^ in 1895 (Figure 1). Current estimates indicate that astrocytes account for ∼20%–40% of brain cells, while glial cells overall may number roughly 50%.^3,4^ This substantial non-neuronal proportion underscores gaps in our understanding of glial functions and ultimately of how the CNS is built and works. It is thus a primary objective in biology and medicine to investigate how glia and other non-neuronal cells interact with and influence neurons and neural circuits, ^5,6^ thereby contributing to physiology and disease. Furthermore, since brain disorders mostly lack adequate disease modifying treatments, there has been growing interest in exploring glia and glia-neuron interaction mechanisms to identify new clinical strategies and targets. Such approaches are needed because drug discovery has proven failure-prone for common CNS disorders. ^7^

Several hagiographies of early glial biology exist, ^1,2^ and, arguably, the key open questions have been appreciated for some time. For example, summarizing his landmark studies with others such as John G. Nicholls that reawakened interest ^2^ in the field, Stephen Kuffler in 1967 wrote^10^

Concerning the role of glial cells, a variety of functions will probably emerge. Obvious gaps in our knowledge, which prevent the formulation of more precise hypotheses relate to detailed information about the biochemical properties of various glial cells and the mechanism of neuron-glia interaction.

**Figure 2.**
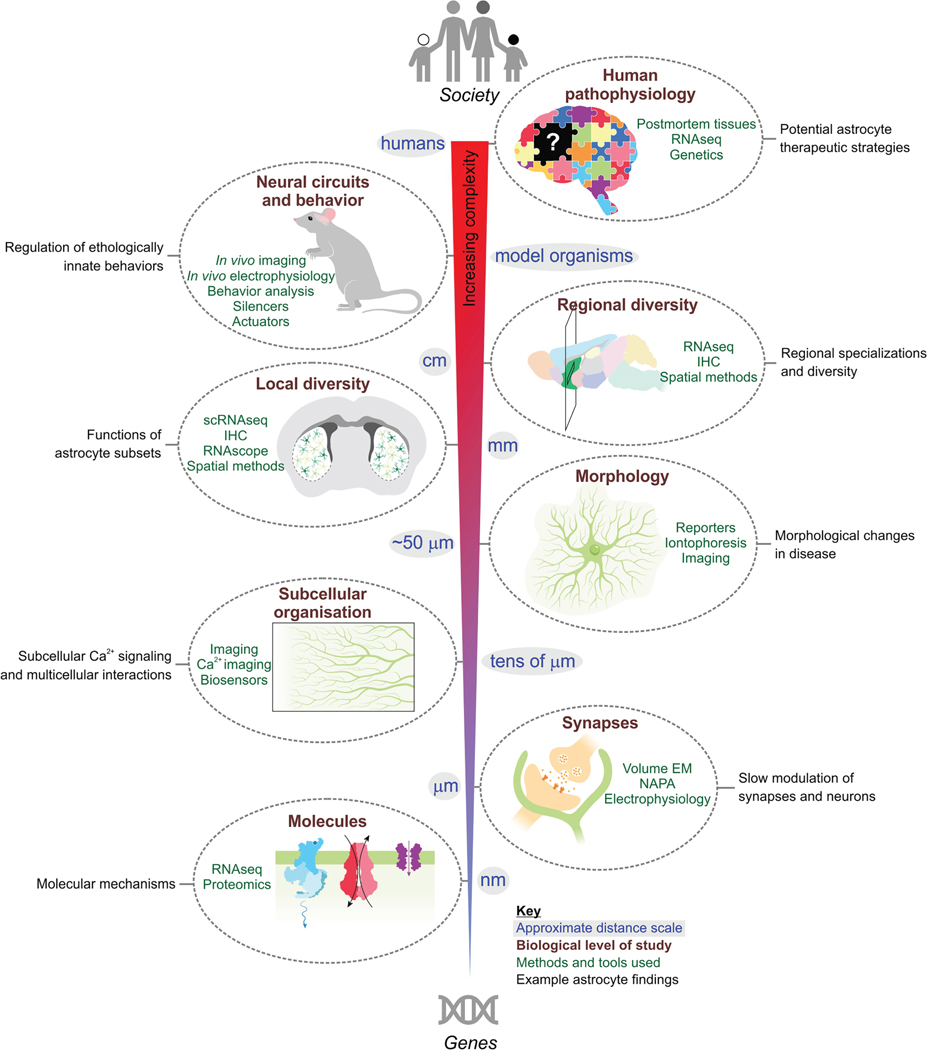
Scales of biology over which astrocytes have been explored Studies across biological scales ranging from the molecular to animals are revealing fundamental biology of astrocyte-neuron interactions. These studies are partly driven by the conviction that there are missing pieces in our understanding of the human brain and its many diseases, with obvious relevance to society. Striatal astrocyte-neuron interactions have been explored across these increasingly complex biological scales, covering a scale from nanometers to humans. Single key findings are listed with a focus on the striatum. Others are discussed in the text. The vertical scale is a schematic and not drawn to accurately represent the distance jumps. The cartoon was inspired by Francis Crick’s “Scales of Biology” frontispiece in What Mad Pursuit. ^12^

In the nearly 60 years since those prescient comments, owing to the efforts of a worldwide community of researchers (see Verkhratsky and Butt ^2^ for review), the field has witnessed important progress in understanding the molecular and biochemical properties of astrocytes. ^11^ However, key questions concerning astrocyte-neuron interactions in mature neural circuits remained either sidelined, unaddressed or incompletely understood. In this review, recent findings on the role of astrocyte-neuron interactions in regulating neural circuits and their contributions to brain disorders are considered at scale (Figure 2). Although the breadth of recent work is covered, the striatum has been chosen for focus for the following reasons.

First, an important realization has been that astrocytes are diverse and differ between and within CNS regions. ^13–15^ This is an important advance but renders problematic conclusions across multiple disorders and diseases that affect different parts of the CNS at different ages, notwithstanding the fact that the molecular causes of the disorders differ. For example, the suggestion that neurotoxic astrocytes kill neurons across many neurodegenerative disorders, ^16^ although valuable and influential as an early idea, is now considered simplistic, ^17^ because astrocytes display variable responses across disorders. ^18,19^ Furthermore, even in a single disorder astrocytes change in separable ways over time and space, as shown in experiments with spinal cord injury. ^20^ In the case of the striatum and intracerebral hemorrhage, astrocyte molecular signatures change in significant ways over several days, and the response to hemorrhage involves multiple cell types, which also vary with respect to distance from the lesion core. ^21^ Similarly, astrocyte Ca^2+^ -dependent vesicular glutamate release is now recognized to perhaps only occur under specific experimental conditions ^22–24^ and only for some small astrocyte subpopulations. ^25,26^ Thus, meaningful exploration of how fundamental properties of astrocytes contribute to physiology and disease requires consideration of specific brain regions, and critically of the neurons and neural circuits there.^5^

Second, although the field lacks complete understanding of astrocyte physiology and pathophysiology in any CNS area, there has been important progress in these regards for astrocytes located within the striatum, which is an important model nucleus for such work. I use such studies to make generally applicable points. This approach does not obviate reviews covering all astrocytes, ^3,5,6,27^ and specific mechanisms and examples permitting conclusions that are germane to other CNS areas are provided.

Third, the striatum is critically involved in normal physiology and in diverse neurological and psychiatric diseases, ^28–32^ rendering it an important nexus to understand astrocyte pathophysiology with relevance to other CNS areas.

Fourth, current estimates suggest that astrocytes evolved around 560–600 million years ago, ^33,34^ which is close in evolutionary time to the emergence of lamprey as the first group of jawless vertebrates that contain basal ganglia (BGs) akin to those of mammals ^35^ and which possess astrocyte-like cells. ^36^ Therefore, exploring the striatum offers insights into how astrocytes and the BGs likely coevolved in circuits controlling basic motor actions fundamental to early life.

Fifth, the astrocyte field is now expansive, rendering it unavoidably superficial to cover everything. Some focus is necessary to provide synthesis and nuance obligatory in a review, and to chart a course for future directions. It has been cogently argued that molecular understanding is necessary for understanding function, ^37^ and accordingly, where possible, molecular mechanisms are highlighted in this review. Some definitions are provided in Box 1.

## ASTROCYTES

An excellent review on the development of astrocytes exists. ^49^ In brief, in mice, cortical astrocytes begin to develop shortly after neurogenesis, around embryonic day 16. Following their migration along radial glia, these astrocytes establish themselves in specific territories, with their properties and locations potentially adapted to meet the needs of neighboring neurons, which may contribute to their brain-region-specific properties. ^50^ In mice, it is estimated that about half of cortical astrocytes arise from local proliferation. ^51^ In contrast, the developmental lineage and anatomical positioning of striatal astrocytes within the striosome and matrix compartments remain incompletely understood, which is also the case for many other CNS nuclei.

### Core functions, electrophysiology, and Ca^2+^ signaling

Astrocytes exhibit several core characteristics, such as complex morphology, that are conserved among species and known to influence neural circuits and brain disorders through diverse mechanisms, many of which are still being investigated. ^5^ Functionally, astrocytes perform a range of tasks, including maintaining ion balance, clearing neurotransmitters, forming and removing synapses, metabolism, modulating synaptic and electrical activity, and participating in neurovascular coupling and the integrity of the blood-brain barrier. ^3^ Since astrocyte processes infiltrate spaces between synapses, they must also restrict diffusion in the extracellular space and thus contribute to the fidelity and specificity of discrete synaptic signals. Astrocytes are also involved in CNS trauma, inflammation, infections, tumors, and both neurological and psychiatric disorders. ^52,53^ The precise ways in which astrocytes contribute to such multifarious physiological and pathological processes are the subject of intense research, with many questions still unanswered.

The field is experiencing a resurgence of interest as advances in methods within neuroscience and glial biology ^54^ have enhanced our understanding of how astrocytes impact brain function. These improvements have enabled researchers to tackle previously challenging questions. For example, although astrocytes do not possess the molecular machinery used to generate Hodgkin-Huxley action potentials, ^55^ recent imaging experiments have revealed small focal (∼20 mV) voltage changes in processes mediated by extracellular K^+^ and glutamate transporter currents ^56^ that may propagate small distances within processes. In relation to this, electrophysiology shows that the membrane potential of astrocytes is stable near the K^+^ Nernst potential due to syncytial coupling, meaning that by post-natal day 15 it is dominated by the membrane potential of its neighbors, ^57^ and further that the low-astrocyte membrane resistance results in a length constant for voltage decay of only ∼3.6 μm. ^58^ Although further studies are needed, it seems likely that small voltage changes in astrocyte processes have local signaling roles, such as regulation of glutamate transport, ^56^ but are unlikely to contribute through propagation over long distances.

The early use ^59,60^ of genetically encoded calcium indicators (GECIs) ^61,62^ to study astrocytes ushered in a new era of experiments and has revealed a rich variety of intracellular Ca^2+^ signals within striatal astrocytes and in astrocytes of other CNS areas, with Ca^2+^ signaling now recognized as a core feature in diverse regions and species. ^63,64^ In contrast to membrane potential fluctuations, intracellular Ca^2+^ signals can be localized microdomains, spread large distances, and cover entire astrocytes. In the striatum, such signals are spontaneous and can be evoked by activation of different neuromodulator receptors on astrocytes. The sources of Ca^2+^ are mainly intracellular, but there is also a measurable transmembrane flux. ^8^ The consequences of triggering and silencing striatal astrocyte Ca^2+^ signaling are considered in later sections. For now, it suffices to note that striatal astrocyte Ca^2+^ signaling is rich and dynamic as in other areas, is a core feature of the cells, and that important progress has been made in exploring its causative roles for neural circuits and behavior (see later sections).

### Metabolism

**Figure 3.**
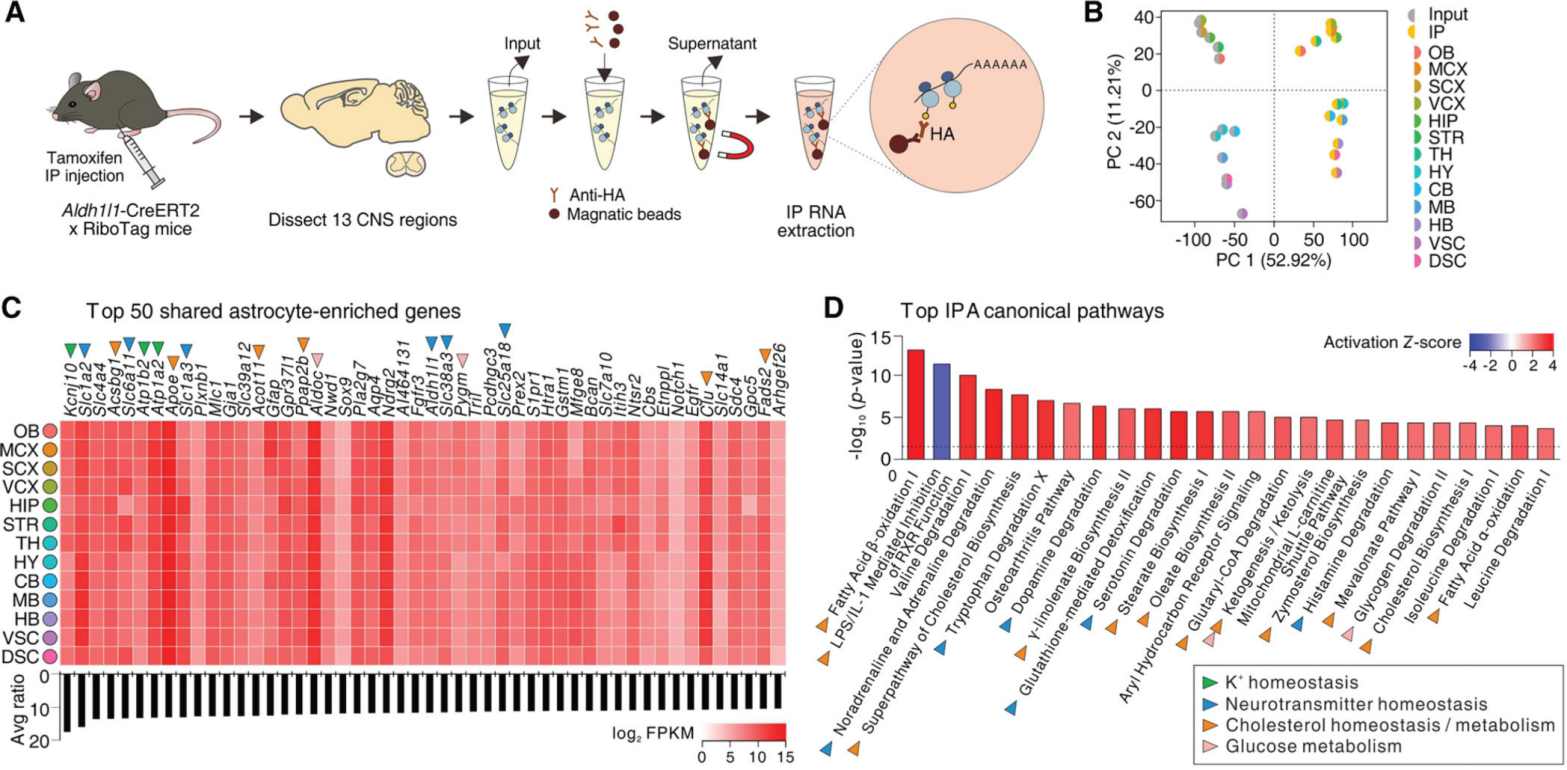
Shared astrocyte CNS molecular signatures and mechanisms from different CNS areas of the mouse that provide clues to how astrocytes regulate neurons (A) Approach for astrocyte-specific RNA-seq using RiboTag mice ^78^ crossed with astrocyte selective *Aldh1l1*-CreERT2 mice. ^79^ (B) Multidimensional scaling plot for input and immunoprecipitated RNA-seq data. (C) Heatmap showing the log_2_ FPKM values of the top 50 most enriched astrocyte genes shared in the 13 CNS regions (CB, cerebellum; DSC, dorsal spinal cord; HB, hindbrain; HIP, hippocampus; HY, hypothalamus; MB, midbrain; MCX, motor cortex; OB, olfactory bulb; SCX, somatosensory cortex; STR, striatum; TH, thalamus; VCX, visual cortex; VSC, ventral spinal cord). The bar graph shows the average ratio of immunoprecipitation (IP) versus input. Colored arrowheads indicate functional class (see D). (D) Bar graph showing the top 25 ingenuity pathway analysis (IPA) canonical pathways for the 825 shared astrocyte-enriched genes across regions with a threshold of *p* value < 0.05. The colored arrowheads indicate functional classes that are mostly related to metabolism. The figure is reproduced from Endo et al. ^50^ and illustrates pathways and genes shared across astrocytes from major CNS regions.

An indispensable role played by astrocytes relates to metabolism, whereby they couple energy supply to neuronal demand to maintain homeostasis and signaling. ^65^ Astrocytes express GLUT1 transporters to import glucose from the blood (via intermediary endothelial cells that form blood vessels). Yet GLUT1 appears not to be the exclusive transporter mediating glucose uptake into astrocytes; its deletion unexpectedly led to an upregulation of glucose uptake and metabolism, suggesting a regulatory role in astrocytic metabolic pathways.^66^ Astrocytic glucose is preferentially metabolized to lactate, which is exported via monocarboxylate transporters (e.g., MCT1/4) and taken up by neurons (e.g., via MCT2) for oxidation in the tricarboxylic acid cycle. This process is called the astrocyte neuron lactate shuttle (ANLS). ^67^ Thus, as the main site of brain glycogen storage, astrocytes mobilize these reserves into lactate to meet the demands of neurons.^68^ Recent evidence also supports a signaling role for astrocyte-derived lactate in regulating neuronal plasticity and gene expression. ^67^ Beyond energy substrates, astrocytes synthesize cholesterol-rich lipoproteins and other lipids vital for synapse formation and myelin maintenance, while bolstering antioxidant defenses by producing molecules such as glutathione precursors, including cysteine. ^69^ In the glutamate-glutamine cycle, astrocytes clear synaptic glutamate via transporters (Glt1, GLAST) and convert it into glutamine for neuronal use. By buffering extracellular ions such as K^+^, they preserve the ionic concentration gradients essential for both metabolism and cellular excitability through Goldman-Hodgkin-Katz considerations. ^70^ Astrocytes are also critical for removal of waste products such as ammonia. Furthermore, through a variety of mechanisms involving vasodilators and vasoconstrictors, astrocytes contribute to neurovascular coupling to dynamically adjust local blood flow in response to neuronal activity. ^71^ By virtue of their endfeet that line the brain’s vasculature, ^72^ astrocytes are also proposed to contribute to fluid flow in the brain through diffusive and directional mechanisms. ^73,74^ Astrocytes also act as sensors of systemic energy availability, integrating metabolic signals such as glucose, lactate, and hormones to adapt their support of neurons accordingly. ^75^ Astrocytic mitochondrial metabolism and redox regulation are increasingly recognized as dynamic modulators of glial-neuronal interactions, influencing not only metabolic support but also inflammation and long-term circuit health. ^76,77^ These fundamental properties of astrocytes are well described and likely shared across brain regions because the molecules that mediate such processes are part of the shared transcriptome of astrocytes across the CNS. ^50^ Bioinformatic analyses of these shared genes also identify metabolism as *the* core function of astrocytes in the striatum and other CNS regions (e.g., K^+^ homeostasis, neurotransmitter homeostasis, cholesterol homeostasis/metabolism, glucose metabolism; Figure 3). In these regards, there appears to be a broad division of labor in the CNS, with astrocytes performing essential metabolic tasks to meet the needs of neurons that are specialized for information processing. In accord, astrocytes express essential molecular machinery needed for metabolism and signaling, whereas neurons express abundantly the molecular machinery needed for electrical excitability, vesicular neurotransmitter release, and fast synaptic transmission.

### Mature morphology

It is easy to imagine how the morphology of astrocytes (Figure 1) caught the imagination of early and subsequent researchers because it differs so much from that of neurons. Each mature astrocyte consists of a cell body, several major branches, larger endfoot bearing branches associated with blood vessels, and numerous finer branchlets and leaflets, often referred to as processes. ^6,80^ While astrocytes have been described as “spongy,” recent high-resolution electron microscopy studies suggest that the alternative “bushy” descriptor may more accurately represent their branching and subcellular organization at the sub micrometer scale. ^40^ Thousands of delicate astrocyte processes interdigitate with other cellular elements of the neuropil, allowing astrocytes to contact all types of CNS cells, including neurons at critical subcompartments such as synapses, axons, and dendrites. ^40,81–83^ However, despite their intricate structure, astrocytes lack the observable clear polarization of neurons, and their complexity is relatively uniform across their compact territories. Such territories minimally overlap in healthy rodent tissue and even when evaluated in some models of trauma. ^84^ The only clearly polarized features of astrocytes appear to be endfeet, which emanate from thicker somatic branches. In the mouse cortex, most astrocytes (99.8%) contact at least one blood vessel, with many forming connections with three, ^85^ and such vascular connections are rapidly repaired following local damage. ^86^ Changes in the morphological features of astrocytes, including alterations in their territories, have been widely reported in disease and aging, ^52^ but the precise consequences remain to be explored mechanistically. Astrocytes are morphologically more complex than in early reports that described them as star-like or spider-like, metaphors that likely reflected limitations of available methods at the time (Figure 1).

### Morphogenesis

Recent studies suggest that astrocyte morphogenesis may be regulated by neuronal activity, ^87^ a variety of mechanisms involving membrane proteins, ^80^ and by self-recognition. ^88^ Additional studies are needed on these alternative or coexisting mechanisms, but from volume electron microscopy work, ^89^ fine astrocyte processes fill all available spaces between neurons, largely lacking self-self contacts and therefore lacking evidence for contact-dependent self-recognition as a phenomenon occurring in mature astrocytes. This may favor the view that neurons direct astrocyte morphogenesis for optimal associations with neurons. In accord, imaging experiments show that astrocyte processes move to produce greater synaptic coverage in response to neuronal activity ^90^ and electron microscopy suggests that active synapses with larger postsynaptic densities have greater astrocytic coverage. ^41^ In cell culture, classic experiments show that astroglia transform from a flattened to a more complex morphology when plated with neurons, ^91,92^ and these findings are widely replicated (see Stogsdill et al.^93^ ). *In vivo*, inhibitory synaptic activity also drives astrocyte morphogenesis. ^87^ Therefore, it seems parsimonious that the complex morphology of astrocytes evolved to compartmentalize and separate adjacent neuronal compartments such as spines and terminals in the extracellular milieu as the complexity of nervous systems expanded. Accordingly, astrocyte morphology may reflect contact-mediated interactions with neurons, in addition to interactions such as self-avoidance between astrocyte processes that may only come into contact with each other during a short developmental window. ^88^ Consistent with this view, the astrocyte-neuron interface is decorated with molecules predicted to mediate cell-cell contacts, including those between astrocytes and neurons.^94^ It is worth noting that it was not possible to image self-self contacts between adjacent astrocyte processes at any developmental stage in the study proposing self-recognition ^88^ and the key molecule studied (Pcdhgc3) regulated astrocyte territory size, which is regulated by many mechanisms, including ion channels, receptors, primary cilia, cytoskeletal proteins, and simply cell health and pathology. ^21,80^ The proposal for self-recognition ^88^ merits further work, and at this stage, it is based mostly on the interpretation of how Pcdhgc3 mutants behave based on prior work with neurons. In these regards, past work on astrocyte neuroligins reported they regulate synapses in a manner analogous to neurons, ^93^ but subsequent studies challenge this idea, ^95^ leaving open what they do in astrocytes. Moreover, Gi-coupled GPCRs hyperpolarize neurons, ^96^ whereas in astrocytes, they cause increases in Ca^2+^ signaling that often acts as an excitability-like signal. ^54^ SAPAP3 in neurons anchors receptors and ion channels to the postsynaptic density, ^97^ but in astrocytes, it regulates the actin cytoskeleton. ^9^ In *Drosophila*, neurons respond to neuromodulators, but astrocytic responses may require prior tyramine receptor activation. ^98^ There are other examples of how a treasured neuronal mechanism does not work similarly in astrocytes. One therefore needs to be thoughtfully circumspect in assigning precise cell biological functions to molecules detected in astrocytes based on expectations from neurons, especially for subcellular phenomena not measured.

### Tiling and coupling

Another enigmatic feature of astrocytes is their tiling of the CNS, ensuring that each astrocyte occupies a distinct territory with minimal overlap with its neighbors. This spatial organization is proposed to be crucial for maintaining overall homeostasis of the CNS and facilitating efficient communication of neurons, for example, by ensuring ionic regulation and by provision of metabolic resources via transport through tiled astrocytes. ^99^ At their boundaries, each astrocyte’s numerous processes interdigitate with those of adjacent astrocytes by no more than about 5% of the total territory, ^100^ which translates to just a few micrometers at the edges. There, they form extensive gap-junctions that enable astrocyte-astrocyte communication and exchange of nutrients, ions, and signaling molecules, and spread of voltage resulting in syncytial membrane potential isopotentiality. ^101^ This tiling arrangement may also be integral to how astrocytes effectively respond to local aberrant neuronal activity and injury. By covering the entire brain in a mostly non-overlapping manner, astrocytes thus play a vital role in ensuring that neuronal populations receive the necessary support for proper brain function and resilience against pathological conditions. Note, however, that studies of the human cortex have shown that astrocytes in the superficial layers display appreciably overlapping territories, which shows that tiling varies between brain regions. ^82^ Furthermore, in the context of injury, astrocytes can undergo dramatic structural changes that alter existing tiling. These include hypertrophy, loss of morphological complexity, atrophy, and extensive reorganization to form glial scars or borders around sites of damage or focal injury. ^52,102^ In this way, the tiling of the CNS by astrocytes is plastic and changes during pathophysiology in a disease-dependent manner. A remarkable feature of astrocytes is the malleability of their responses, which are context- and disease-specific when assessed at a molecular level. ^18,19^ The full implications of context-specific astrocyte responses merit further study, including from the perspective of tiling.

### Other species

Human and non-human primate astrocytes are larger than their mouse counterparts, with each human astrocyte territory encompassing around 2 million excitatory synapses. ^103^ In contrast, a rat hippocampal astrocyte typically contains about 100,000 excitatory synapses, ^100^ although this frequently cited number should be considered thoughtfully, as the number of synapses will vary by brain region and species; e.g., a mouse striatal astrocyte territory contains ∼56,000 excitatory synapses. ^8,104^ The presence of other types of neurotransmitter and neuromodulator synapses within astrocyte territories are not well documented. The molecular basis and functional implications of the larger size of human astrocytes are still being explored; however, recent studies indicate that while astrocytes from non-human primates are larger than those in mice, they do not exhibit much greater morphological complexity when analyzed carefully. ^105^ This finding suggests that the complexity of astrocytes may have simply scaled with their size. Astrocytes and astroglia from the fruit fly and zebrafish, respectively, also display morphological complexity ^106,107^ but are smaller than those of mice or primates. Furthermore, although varicose projection astrocytes were considered a uniquely hominid feature, ^103,108^ recent work shows that they may reflect a form of reversible reactivity and are observed in other species and disease states.^109^ Detailed studies exploring human astrocytes in postsurgical tissue slices or in 2D and 3D cell culture models are much needed.

## STRIATAL ASTROCYTE-NEURON INTERACTIONS

Advances have occurred in our understanding of how astrocytes regulate neurons and neural circuits with the striatum showing value as a model nucleus ^110^ to tackle the broader question of how astrocyte-neuron interactions affect circuits and behavior. The application of GECIs, ^111^ genetic tools such as designer receptors exclusively activated by designer drugs (DREADDs), ^96^ and gene deletion experiments have led to important advances in understanding the functions of astrocytes within the striatum. ChannelRhodopsin is less useful for astrocytes ^112^ because it elevates extracellular K^+^ levels that have well known effects on all cells, including neurons.

### Core features of the striatum in relation to astrocyte-neuron interactions

The striatum is the largest nucleus within the BGs, which is a group of interconnected subcortical nuclei that play crucial roles in essential functions, including movement, action selection, reward processing, addiction, and the manifestation of a wide range of neurological and psychiatric disorders. ^29^ In rodents, approximately 95% of striatal neurons are classified as medium spiny neurons (MSNs; also called spiny projection neurons or SPNs), which are γ-aminobutyric acid (GABAergic) projection neurons marked by the presence of DARPP32 proteins. ^113,114^ The striatum also contains GABAergic and cholinergic interneurons (ChIs). The striatum receives a significant amount of excitatory input from the cortex and thalamus, specifically targeting the dendritic spines of MSNs that express either D1 or D2 dopamine (DA) receptors, i.e., D1 and D2 MSNs. Additionally, the striatum is influenced by extensive input from midbrain DA neurons originating in the substantia nigra pars compacta and the ventral tegmental area (VTA). One of the primary functions of the striatal circuitry is to determine which subpopulations of MSNs will fire action potentials from their normally hyperpolarized states in response to coordinated inputs that may give rise to UP states. ^115^ This activity is essential for shaping the output of downstream nuclei, thereby regulating behaviors controlled by the BGs. ^32^ In a simplified model of striatal function, activation of D1 MSNs inhibits the firing of BG output nuclei, such as the substantia nigra pars reticulata (SNr) and the internal globus pallidus (GPi), through a pathway known as the direct striatonigral projection. This inhibition leads to the disinhibition of thalamocortical circuits, facilitating movement. Conversely, activation of D2 MSNs enhances the activity of the SNr via the striatopallidal (indirect pathway) circuit, which results in the inhibition of thalamocortical drive. However, the roles of MSNs are far more intricate than this simplified dichotomy suggests, and ongoing research continues to explore these complexities. Furthermore, the striatum itself is large and comprises several functional and anatomical subregions, ^114^ receiving and making distinct complements of afferent and efferent connections associated with different aspects of behavior such as motivation, action selection, and learning. For example, the dorsolateral striatum (DLS) is primarily involved in motor output and habitual behaviors, while the ventral striatum (VS), including nucleus accumbens (NAc), is involved in encoding motivated behaviors and facilitating goal-directed learning. The striatum is not a layered structure like the hippocampus or cortex, and functional and anatomical subregions are not clearly defined. Instead the striatum is intermixed with cell types and connections, but interleaved within this disordered structure are labyrinth-like specialized areas in the form of striosome and matrix compartments that are revealed with appropriate markers for genes, biochemistry, and connectivity. ^30,114,116^ Astrocytes interact spatially with D1 and D2 MSNs throughout the regions of the striatum, but whether this differs between striosome and matrix compartments is unknown. A *Crym*-positive astrocyte subset is slightly more dominant in matrix than striosome compartments from immunohistochemical evaluations. ^117^

In addition to its significant relevance, the striatum exhibits characteristics that render it ideal for investigating mechanisms by which astrocytes modulate neural circuits. First, the striatum constitutes substantial mouse brain volume, providing sufficient size to facilitate physiological and biochemical investigations both *in vitro* and *in vivo*. Second, the neuronal population within the striatum mostly comprises MSNs, which is advantageous for studying astrocytes without the attendant complexity of dealing with comparatively incompletely characterized neuronal populations in other parts of the brain. Third, the principal inputs and outputs of the striatum are well characterized, allowing for the functional roles of astrocytes to be effectively benchmarked against their impact on core striatal physiology. Lastly, given the striatum’s involvement in various diseases and addiction, the fundamental biology of astrocyte-neuron interactions within this region may offer valuable insights for therapeutic exploitation.

### Striatal astrocyte molecular identity based on gene expression

Extensive work ^8,104,112,118,119^ on the basic electrophysiological properties of striatal astrocytes demonstrated that they share passive features with astrocytes elsewhere in the CNS. Furthermore, intracellular Ca^2+^ signals of striatal astrocytes were broadly similar to astrocytes elsewhere in the CNS. ^120^ Thus, two widely used functional methods did not reveal unique features of striatal astrocytes, although some differences were noted. ^8^ However, owing to the range of parameters that can be assessed and compared, the use of deep RNA sequencing (RNA-seq) using RiboTag methods ^78^ provided evidence that astrocytes from the striatum were separable from those in other parts of the CNS. ^8,50^ Furthermore, astrocyte diversity between different brain regions has since been documented in large-scale brain cell atlas projects for mice and humans. ^121–125^ Functional studies comparing astrocytes systematically between brain regions are now needed to provide physiological context to diversity.

By comparing astrocytes from 13 CNS regions with RNA-seq, ∼4,300 astrocyte-enriched genes were identified and of these ∼20% (825) were shared (Figure 3). Half the pathways and signaling mechanisms shared by astrocytes across all regions represented by these 825 genes were related to metabolism, enzymatic and transporter activity, or transcriptional regulation. More specific analyses showed that for K^+^ homeostasis, the most highly expressed genes were *Kcnj10*, *Kcnj16*, and *Atp1a2*, encoding K^+^ channels and Na^+^ /K^+^ ATPase. Astrocytes also highly expressed genes related to neurotransmitter transport and metabolism, but they lacked or expressed at very low-level genes related to Ca^2+^ -dependent vesicular glutamate release. ^50^ The top two genes shared across astrocytes were *Kcnj10* (Kir4.1) and *Slc1a2* (Glt1). Overall, core shared features between large parts of the CNS include K^+^ homeostasis, neurotransmitter homeostasis, cholesterol homeostasis/metabolism, glucose metabolism, and fatty acid β-oxidation ^50^ (Figure 3). Much further work is needed to understand the core functions of astrocytes across CNS areas, but these evaluations show that homeostasis and metabolism are shared features of astrocytes across the CNS.

By analyzing astrocyte genes in 13 CNS areas relative to the other 12 regions, 3,500 region-enriched genes were identified that clustered with anatomical proximity of the regions relative to each other. Striatal astrocytes clustered with the olfactory bulb, motor cortex, sensory cortex, visual cortex, and hippocampus. ^50^ The number of region-enriched genes varied for astrocytes across regions and mainly comprised upregulated genes, with the correlation analyses of the data suggesting that regional identity of astrocytes may reflect both the region-specific microenvironment and astrocyte-lineage-related cues such as transcription factors. ^50^ This duality between extrinsic and intrinsic factors determining astrocyte identity has been explored for the septum by cell transplantation experiments ^126^ and is consistent with the finding that activity regulates astrocyte morphogenesis. ^87^ However, further studies are needed to directly explore whether mature astrocyte identity is determined most by local neuronal effects or by developmental lineage-related cues. RNA-seq analyses also identified region-specific astrocyte marker genes for each of the 13 CNS areas, with *Crym* being the top gene that separated striatal astrocytes from those in other areas. Furthermore, when assessed by scRNA-seq, astrocytes within the striatum comprised seven discernible subclusters. ^18,50^ Overall, the data provided strong evidence to indicate that striatal astrocytes have specific molecular features and functions and that these functions may arise from the local environment and by variable representation of smaller subclusters of astrocytes in a region-specific manner. While the allocation and specific roles of region-specific astrocyte subclusters in the striatum remain to be fully studied, recent work has explored one striatal astrocyte subpopulation defined by *Crym*. ^117^ It seems likely that the functions of the striatum may be locally regulated by specific populations of astrocytes, while critical core functions are shared across astrocytes.

### Striatal astrocyte subproteomes

Analysis using designer tools to examine striatal astrocytic and neuronal subproteomes demonstrated that gene expression unreliably predicts protein levels, except in cases of highly expressed proteins. ^9^ This was to be expected from past work and does not detract from the use of RNA-seq to classify cells but instead portends mechanisms possibly missed by gene expression evaluations alone. Comparative analysis of striatal astrocytic and neuronal proteomes revealed both common and unique signaling pathways specific to each cell type. ^9^ While further investigation is needed, the study of astrocyte subproteomes from regions, such as endfeet and areas involved in glutamate and K^+^ regulation, has provided insights into how these morphologically complex cells function across different domains to mediate distributed physiology. ^9^ An important finding was the preferential concentration of proteins within specific astrocyte subproteomes, which has implications for understanding striatal pathophysiology. This finding suggests that astrocytes organize their critical functions into specific domains within their complex structures ^9^ –which is consistent with their complex morphology. The identification of these subcompartment proteomes has unveiled previously unknown molecules and pathways in astrocyte physiology. Moving forward, research in this field will likely benefit from utilizing a combination of viral vectors ^9^ and transgenic mouse models ^127^ to study astrocyte and MSN subproteomes. Furthermore, these approaches need to be applied to astrocytes throughout the CNS. Interestingly, an earlier evaluation of isolated astrocytes provided proteomic data supporting differences of striatal and hippocampal astrocytes, ^8^ but proteomic methods have not yet been used to study astrocyte diversity more broadly. In later sections, two proteins identified within striatal astrocyte subproteomes (μ-crystallin and SAPAP3) are discussed from the perspective of how they contribute to astrocyte-neuron interactions.

### Striatal astrocyte markers

A consistent finding from gene and protein expression evaluations is that glial fibrillary acid protein (*Gfap*, GFAP) is not highly expressed in striatal astrocytes under normal conditions. This has implications for the analysis of human postmortem striatal tissue because such studies often use GFAP as a marker. In such studies, striatal astrocytes may be more reliably identified by S100β, Aldh1l1, GLT1, and Sox9 antibodies. Other highly expressed striatal astrocyte proteins such as μ-crystallin, Kir4.1, and GAT3 can also be used with the qualification that they may display cell-to-cell expression variability or be expressed in subpopulations of astrocytes. It is interesting to note that astrocyte-like cells from lamprey ^128,129^ also lowly express GFAP and instead use members of the Cytokeratin family as their intermediate filaments. The relative lack of GFAP in striatal astrocytes may be an ancient conservation and it has been proposed based on evolutionary analysis that lack of GFAP may confer functional benefits on some astrocytes. ^130^

### Striatal astrocytes and neuronal investment

Striatal astrocytes are morphologically complex, compact, and bushy when imaged with cytosolic reagents such as GFP, lucifer yellow, or tdTomato ^8,79,120^ (Figure 1). However, with cell surface reporters, they appear as fluffy cloud-like cells ^79^ because the finest astrocyte processes with dimensions on the tens of nanometer have high surface areas and low internal volumes. Serial block face scanning electron microscopy (SBF-SEM) shows that such fine striatal astrocyte processes do not contain abundant vesicles, ^8^ which is in accord with cortical astrocytes observed with SBF-SEM and focused ion beam SEM (FIB-SEM). ^40,42^ Classical markers such as Kir4.1, GAT3 and Glt1 are generally evenly expressed within striatal astrocytes and not restricted to subcellular structures detected with light microscopy implying that synapses and sites of homeostasis exist within entire astrocytes. In contrast, Aqp4 is highly enriched in endfeet, underscoring the polarized nature of that compartment.^9^ Striatal astrocytes display differences and some similarities with astrocytes elsewhere in the CNS, for example, in territory size, that supports astrocyte diversity between CNS regions.^50^ The exact relationship between astrocyte territory size and function is not well understood, and territory size may reflect neuronal requirements within brain regions.

In the DLS there are about six times more neurons than astrocytes, ^8^ with each astrocyte contacting ∼11 MSNs (∼6 D1 MSNs and ∼5 D2 MSNs). ^104^ The finding that striatal astrocytes contain within their territories many MSN somata suggests that they may regulate composition of the extracellular milieu around neurons and thus their excitability. ^112,119^ Furthermore, since a core function of astrocytes is to provide metabolic and homeostatic support to neurons, then such functions come into sharp focus within the striatum where the neuron:astrocyte ratio is high. It remains to be determined whether such high demand on astrocytes contributes to selective vulnerability of the striatum in neurodegenerative diseases. A single MSN has dendrites that arborize locally to an area of ∼400 μm diameter in a plane, which would include ∼170 astrocytes in 3D, ^104^ but the analysis of astrocytic interactions with dendrites is still a nascent area and requires methods specific to those contacts. Single striatal astrocyte territories contain ∼50,700 excitatory synapses with astrocyte process-synapse distances of around 50 nm (range 10–400 nm) indicating that astrocyte leaflets interact with excitatory synapses at multiple ranges, recalling findings in the hippocampus. ^41^ In contrast proximity of astrocyte processes to dopaminergic terminals was generally longer at ∼250 nm. ^104^ Together, these data show that astrocyte processes make variable finger-like associations with synaptic structures. Furthermore, within a single astrocyte, the density of inputs largely followed the complexity of the imaged astrocyte, implying that distinct inputs may not sample anatomically observable astrocyte subdomains. Altogether, these data suggest, as mentioned earlier, that elaborate astrocyte morphology may fill available spaces and biochemically compartmentalize synapses or sets of synapses from the extracellular space and from each other. Whether synapse specificity of astrocytic interactions occurs at the level of highly localized receptor signaling remains to be properly explored but may be suggested based on interpretations of electrophysiological data. ^131^ Super resolution methods, perhaps combining light with electron microscopy, may be useful here. Furthermore, although higher resolution volume electron microscopy data are much needed, there is no obvious evidence for striatal astrocyte processes from the same cell extensively contacting each other in any of the available light microscopy data.

### Electrophysiology

Functionally, striatal astrocytes appear similar to the astrocytes in other brain areas, and electrophysiological differences between striatal and hippocampal astrocytes were subtle. ^8^ Striatal astrocytes display linear current-voltage relations, negative resting membrane potential (Vm) of about − 85 mV, and low membrane resistances of less than 10 MOhm. Block of Kir4.1 channels depolarized the membrane only by a few millivolts, implying that other K^+^ channels, whose identities are currently undefined, must keep the Vm close to the K^+^ equilibrium potential. ^112,119^ Gap-junctional coupling between astrocytes in the striatum was extensive and likely confers isopotentiality on the astrocyte syncytium as well as allowing exchange of solutes between hundreds of coupled cells. ^132^

### Ca^2+^ signaling events

Utilizing various types of GECIs revealed that striatal astrocytes exhibit complex intracellular Ca^2+^ signaling patterns, akin to those observed in astrocytes from other brain regions and described as a sparkling panorama. ^59^ Thus, striatal astrocytes display extensive intracellular Ca^2+^ elevations, which are driven by Ca^2+^ entry across the plasma membrane, by Ca^2+^ release from intracellular stores and by neurotransmitters and neuromodulators. ^8,79,120,133^ Generally, three distinct types of spontaneous Ca^2+^ signals have been identified: (1) global waves that encompass the cell body and large portions of astrocyte branches, (2) local waves that are confined to smaller segments of the branches, and (3) frequent microdomains that cover only micrometer-scale areas of branches and cell bodies. ^120^ These Ca^2+^ signals result from transmembrane Ca^2+^ fluxes, release of Ca^2+^ from intracellular stores, ^8^ and are typically slow and can last several seconds or longer. Fast signals have not been observed in striatal brain slices,^120^ but may be expected to occur *in vivo* during behavioral paradigms based on studies of the cortex. ^134^ Waves of intracellular Ca^2+^ traveling between astrocytes of the striatum have not been observed, but the possibility that a Ca^2+^ event in one astrocyte increases the probability of an event occurring in a neighboring astrocyte needs to be explored. Existing reviews cover astrocyte Ca^2+^ dynamics, and this topic is not covered further. ^63,64,135,136^

One challenge specific to the striatum for imaging is its deep location and the fact that it receives substantial input from the cortex. This dense cortical input complicates *in vivo* studies of striatal astrocyte Ca^2+^ signaling because removing the overlying cortex for *in vivo* imaging eliminates a major physiological input. Additionally, implanting lenses or cannulas into the striatum for optical access causes astrocytes to become reactive because of the foreign body response, ^137^ almost certainly affecting the results owing to the diverse consequences of reactive astrogliosis ^17^ on neurons. To avoid these complications, much of our knowledge about *in vivo* astrocyte Ca^2+^ signaling comes from studies of astrocytes in the superficial layers of the cortex. It is likely that the general features of astrocyte Ca^2+^ signals ^63,64^ are similar between cortex and striatum, but that specializations reflecting the local neuromodulatory environment of the striatum have not yet been adequately studied *in vivo*.

One study demonstrated that astrocytes located in the dorsal striatum exhibit distinct functional interactions with D1 and D2 MSNs. ^131^ Although there are caveats, ^110^ this finding implies that subsets of astrocytes operate in a microcircuit-specific manner, which may have implications for the functioning of the striatum and the behaviors it encodes such as movement, decision-making, and reward processing. However, establishing a clear connection between the physiological characteristics of astrocytes and specific behavioral outcomes has proven to be a more difficult challenge. This complexity arises from the multifaceted roles that astrocytes play in neuronal communication, as well as the dynamic nature of their interactions with MSNs. Consequently, researchers have used innovative experimental techniques and methodologies to explore these relationships effectively both in brain slices and *in vivo* ^54^ in adult mice.

### GPCRs and Ca^2+^ signaling

**Figure 4.**
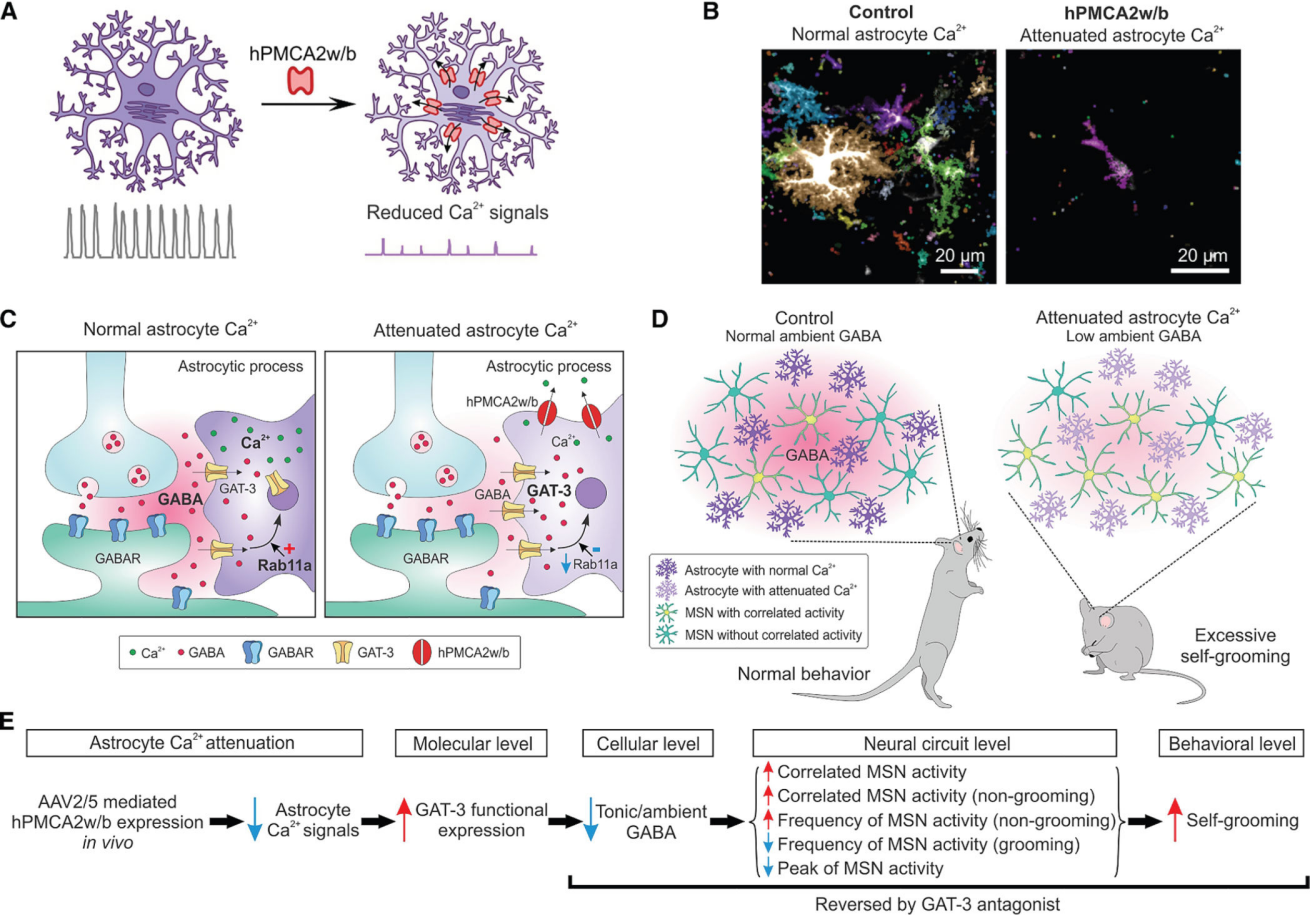
Summary of how attenuating striatal astrocyte Ca^2+^ signaling *in vivo* altered innate behavior to cause excessive self-grooming (compulsive-like behavior) (A) The cartoon summarizes how hPMCA2w/b works to reduce astrocyte intracellular Ca^2+^ signals: it works as a pump (hPMCA2w/b is also called CalEx). (B) z stack images of intracellular Ca^2+^ imaging from striatal astrocytes expressing hPMCA2w/b or a control protein. Spontaneous Ca^2+^ events detected by AQuA were labeled in different colors. (C–E) The cartoon summary is of the main findings at synaptic (C) and *in vivo* levels (D). (E) Description of the proposed sequence of events. In brief, attenuation of striatal astrocyte Ca^2+^ signals reduced Rab11a, which resulted in increased GAT-3 functional expression. This reduced ambient γ-aminobutyric acid (GABA) levels in the extracellular space and tonic inhibition. The data are consistent with a model in which reduced tonic inhibition alters MSN firing and downstream circuits to cause excessive self-grooming. In accord, tonic inhibition and self-grooming were rescued by a GAT-3 antagonist. These findings summarize experiments showing how silencing astrocyte Ca^2+^ signaling *in vivo* affects neurons, neural circuits and innate behavior via a GAT-3 dependent mechanism regulating tonic neuronal inhibition. This figure is assembled from Yu et al. ^133,138^

Recent research has established intriguing connections between astrocytic Ca^2+^ signaling and alterations in animal behavior, sometimes mirroring phenotypes observed in mouse models of various human psychiatric disorders. Astrocytes in the striatum, similar to those in other regions of the CNS, express a variety of G-protein-coupled receptors (GPCRs). Among these, GABA_B_ GPCRs occupy an important role because striatal MSNs are GABAergic and activation of GABA_B_ receptors on astrocytes leads to an increase in intracellular Ca^2+^ levels.^8^ To begin investigating the *in vivo* functions of astrocytic Ca^2+^ signaling, a strategy to attenuate Ca^2+^ signaling by heterologous expression of a specific Ca^2+^ pump known as CalEx (hPMCA2w/b) was developed^133,138^ (Figure 4). This pump functions as a Ca^2+^ exporter, effectively reducing intracellular Ca^2+^ signals in astrocytes by more than 80%. Mice with significantly diminished striatal astrocyte Ca^2+^ signaling displayed repetitive, compulsive-like behaviors, such as excessive self-grooming.^133,138^ These behavioral changes were associated with GABA-mediated alterations in tonic inhibition, which were further modulated by the astrocytic GABA transporter (GAT), GAT-3. In stark contrast, a global brain-wide attenuation of astrocytic Ca^2+^ signals–achieved through the same CalEx mechanism–resulted in premature lethality.^138^ This outcome suggests that astrocytic Ca^2+^ signaling facilitates several functions beyond those observed in the striatum, highlighting its critical role in maintaining overall brain homeostasis and metabolism. However, it is crucial to note that the underlying mechanisms may differ across brain regions, because the attenuation of astrocytic Ca^2+^ signaling influenced behavior and impacted gene expression.^138^ Additionally, the nature of how CalEx works means that it affected many forms of astrocytic Ca^2+^ signaling, without the capacity to differentiate between spontaneous signals and those that are mediated by neuromodulators and neurotransmitters. Given these complexities, while CalEx serves as a valuable experimental tool, its application must be complemented by direct investigations into the specific mechanisms that link altered astrocyte activity to altered neuronal functions. An example of this is seen in the GAT3-dependent modifications of striatal tonic inhibition,^133^ which highlights the need for multifaceted approaches. The evidence suggests that while CalEx can attenuate astrocyte intracellular Ca^2+^ signals, it does not markedly elevate Ca^2+^ levels in the comparatively much larger extracellular space.^133,139^

The use of CalEx within striatal astrocytes allowed the first direct link to be made between astrocyte Ca^2+^ signaling, altered neuronal function, altered neural circuit dynamics *in vivo*, and an ethologically innate behavior in a way that was also reversed by blocking GAT-3, the relevant mechanism suggested by molecular studies^133^ (Figure 4). This was a relevant conceptual advance showing astrocyte signaling alters neural circuits and shapes behavior. The CalEx approach has subsequently been used successfully in a variety of other astrocyte studies such as for compulsive/perseverative behaviors,^140^ neurovascular coupling,^141,142^ reward-cue associations,^143^ synaptic modulation,^144^ cocaine seeking,^139^ zebrafish passivity behaviors,^145^ and others,^146–150^ including studies of Ca^2+^ dynamics in microglia^151,152^ and oligodendrocytes.^153^

Since GABA_B_ receptors are Gi coupled, functions of this GPCR signaling pathway were explored in astrocytes from the dorsal striatum by activation of hM4Di DREADDs. A DREADD approach was used because GABA_B_ receptor agonists would not be able to selectively activate astrocytic GABA_B_ receptors. Astrocytic hM4Di activation triggered upregulation of the gene for a synaptogenic cue, ^154^ thrombospondin-1 (TSP-1). This in turn resulted in enhanced excitatory synaptic transmission onto MSNs, increased neuronal firing, and behavioral phenotypes related to hyperactivity with disrupted attention that were reversed by gabapentin that blocks the TSP-1 receptor α2δ−1 on neurons. ^154^ Physiologically, the data are consistent with the hypothesis that GABA is released from MSNs and activates Gi-coupled GABA_B_ GPCRs, which are highly expressed in astrocytes. Located deeper in the NAc, astrocytes in the VS responded to DA release from VTA neurons by elevating intracellular Ca^2+^ levels.^155^ When DA D1Rs or intracellular IP_3_R2s were deleted from astrocytes in the NAc, the mice displayed dampened locomotor responses to amphetamine, suggesting involvement of NAc astrocytic Ca^2+^ signaling in reward and addiction behaviors. Furthermore, cocaine increased NAc astrocyte Ca^2+^ signaling, whereas attenuating astrocyte Ca^2+^ signaling decreased the number of silent synapses in the NAc shell in response to cocaine. Such mechanisms, operating via TSP 2, contribute to cue-induced cocaine seeking after withdrawal, and cue-induced reinstatement of cocaine seeking after extinction.^156^ Overall, these studies highlight the signaling interplay between astrocyte GPCRs, Ca^2+^, and molecules with known synaptogenic effects.

In a subsequent study^157^ a peptide called iβARK, which is derived from β-adrenergic receptor kinase 1 was introduced. This 122-residue inhibitory peptide was specifically designed to attenuate Gq-GPCR signaling in astrocytes, allowing for targeted exploration of the behavioral effects and physiological functions mediated by this astrocytic signaling pathway *in vivo*. The effectiveness of iβARK was demonstrated through its ability to significantly reduce Gq GPCR-mediated Ca^2+^ signaling *in vitro* and *in vivo* without impairing spontaneous Ca^2+^ signaling, cellular morphology, or gene expression in astrocytes. Moreover, iβARK attenuated behavioral hyperactivity caused by activation of the striatal Gq-GPCR pathway with hM3Dq DREADDs, and it attenuated startle-evoked responses in cortical astrocytes known to be mediated by noradrenaline acting on Gq-coupled astrocytic GPCRs. ^157^ By deploying iβARK brain wide, the authors found that attenuation of Gq-GPCR signaling resulted in changes in mouse behavior, particularly in tasks relevant to cognitive processes related to behavioral adaptation and spatial memory. ^157^ The study highlighted that iβARK fine-tunes astrocyte responses to behavioral stimuli while not interfering with other GPCR-mediated pathways. This specificity is crucial, as past approaches to studying astrocytic signaling were often complicated by the overlapping effects of GPCR signaling present in multiple cell types. iβARK promises to be a useful tool to explore Gq-GPCR signaling and has been used for such purposes in other brain areas and cells ^144,158,159^ but could also be improved to make it light or drug-activatable. There is also a need for similar approaches for the Gi and Gs GPCR pathways. Perhaps expression of pertussis toxin reported in *Drosophila* astrocytes ^98^ may be similarly effective in vertebrates.

### Astrocytic regulation of striatal DA

DA release in the striatum is crucial for numerous brain functions, including making choices, motivation, movement, and reward. Dysregulation of DA release is associated with disorders such as Parkinson’s disease, schizophrenia, and addictive drug action. GABA plays a significant role in inhibiting DA output by acting at GABA_A_ and GABA_B_ receptors thought to be located on DA axons.^160^ Regulation of DA release by GABA depends on the types and locations of these receptors, as well as the sources of GABA in the striatum, which include local interneurons, GABA co-release from DA axons and the overall level of ambient GABA, which is shaped by GATs including those on astrocytes (more details on this in the section about Parkinson’s disease). In addition, adenosine A1 receptors (A1Rs) inhibit DA release in the NAc core (NAcC) by responding to ambient adenosine levels. ^161^ These levels are regulated by astrocytic equilibrative nucleoside transporter 1 (ENT1). When A1R agonists are introduced, they lead to a significant decrease in evoked DA release. ^161^ The importance of ENT1 in this process is notable, as inhibiting ENT1 raises ambient adenosine levels, which further increases A1R-mediated inhibition of DA release. The study demonstrated that changes in ENT1 function directly on astrocytes–whether through pharmacological means or targeted genetic modification–affect DA release. ^161^ This indicates that astrocytes play a vital role in regulating striatal adenosine levels, which are necessary for maintaining proper DA output. Additionally, this link between astrocytic ENT1, adenosine, and DA provides a bridging mechanism for how acute exposure to ethanol affects DA dynamics. It was found that ethanol raises extracellular adenosine levels by impairing ENT1 function, which leads to increased tonic inhibition of DA output. ^161^ This finding helps explain how ethanol influences motor coordination and reward processing, offering valuable insights into substance use disorders and highlighting the potential role of astrocytes in regulating DA function in the striatum.

### Satellite astrocytes, ChIs, and DA release

**Figure 5.**
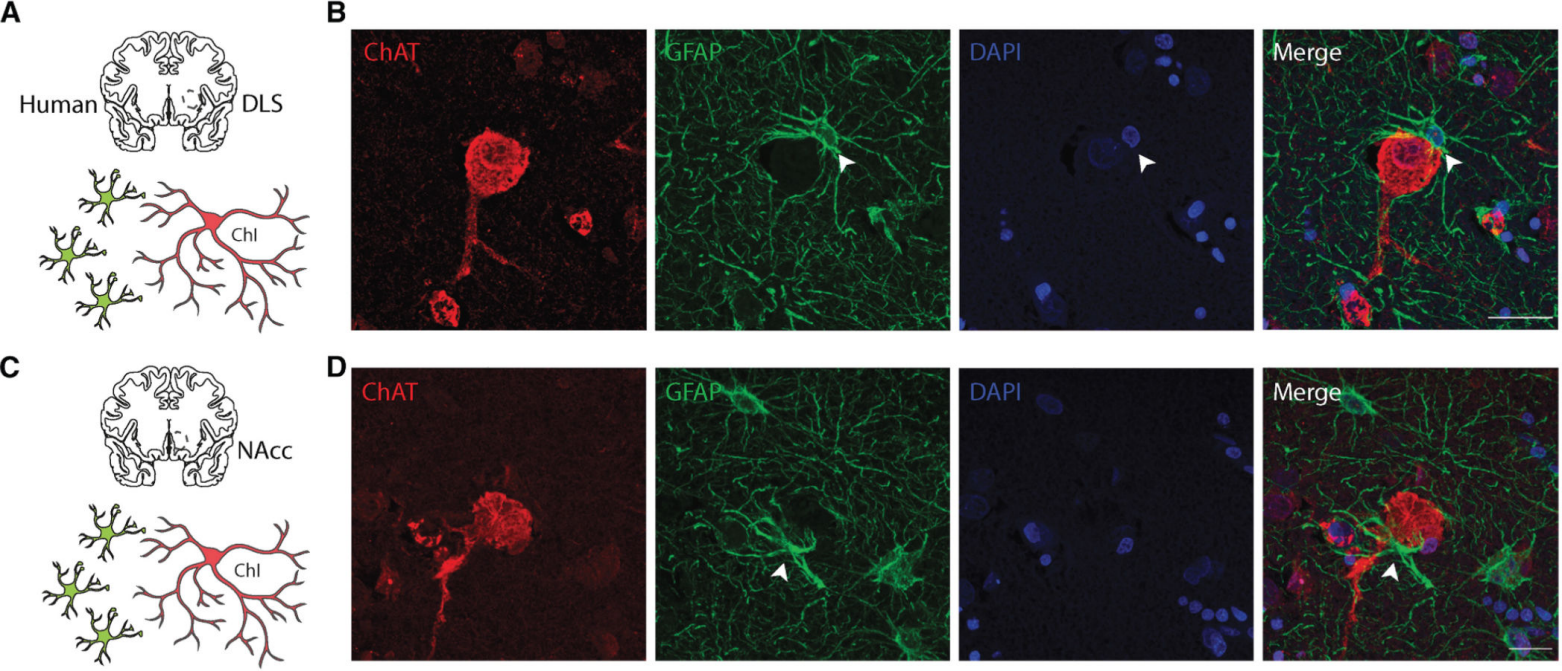
Human ChIs form soma-soma appositions with satellite astrocytes (A and C) Schematics of anatomical areas. (B and D) Maximum projection confocal images showing human ChIs (labeled with antibodies against ChAT; red), closely accompanying and ensheathing astrocyte (labeled with GFAP antibodies; green; arrowhead), and DAPI+ nuclei (blue) in (B) human putamen and (D) human NAcc. Scale bar, 20 μm. Figure is reproduced from Stedehouder et al. ^162^

It was recently found that a population of striatal astrocytes occupy a unique anatomical arrangement whereby they adopt a “satellite” position around ChIs with a soma-to-soma configuration between such satellite astrocytes and ChIs ^162^ in mice and humans (Figure 5). The percent of ChIs with soma-to-soma contacts with satellite astrocytes was ∼43% in NAc and ∼20% in DLS. ^162^ This specific anatomical arrangement presumably allows astrocytes to rapidly regulate neuronal activity, facilitating communication and signaling between the cells. Transient astrocyte depolarization by just a few millivolts led to rapid changes in extracellular Ca^2+^ levels, which altered the firing rate of adjacent ChIs in complex ways with short and long latencies (both increases and decreases were observed). Precisely how Ca^2+^ levels change in response to physiological activity of satellite astrocytes and how these affect the ion channels controlling ChI firing remains to be fully elucidated, although multiple plasma membrane Ca^2+^ channels or Ca^2+^ -gated cation channels are credible candidates–nonetheless the phenomenon was elegantly demonstrated. ^162^ Furthermore, the ensuing changes in acetylcholine (ACh) regulated the release of DA via presynaptic nicotinic ACh receptors that are known to potently affect DA release. Overall, the mechanism is fascinating as it shows the direct influence astrocytic activity has on the excitability of ChIs through an ion homeostasis mechanism that operates on rapid sub second time scales. ^162^ Additionally, the researchers sought to establish the translational relevance of their findings by examining human postmortem brain tissue. They performed immunofluorescent staining which revealed a similar satellite organization of astrocytes around ChIs in the human striatum, indicating that this regulatory interplay between astrocytes and cholinergic neurons is not limited to rodent models but is also conserved in humans (Figure 5). The results of this study underscore the importance of astrocytes as active modulators of striatal circuit function by regulation of extracellular ionic composition, which was previously shown ^119^ for K^+^. The findings have implications for understanding neurological and psychiatric disorders where dopaminergic and cholinergic systems are disrupted or can be usefully targeted e.g., in Parkinson’s, dystonias, schizophrenia, Huntington’s, further highlighting the potential of astrocytes as therapeutic targets in conditions characterized by altered striatal signaling.

### Astrocyte ensembles in the NAc

By adopting a method to genetically label cells showing elevations of intracellular Ca^2+^ via an approach called Cal-Light, ^163^ a recent study succeeded in adapting the methodology for astrocytes to identify ensembles activated during cue-reward associations using the approach they called AstroLight. ^143^ This ensemble was an astrocyte subset within the NAc, and reactivation and silencing of it with genetic approaches modulated cue-reward associations. The study provides both a new tool to identify and manipulate astrocytes ensembles *in vivo* but also shows that functionally identified astrocyte subsets serve specific roles for behavior. This is important because it opens the possibility of identifying astrocyte subsets based on functional criteria (“ensembles”) rather than criteria based on gene expression alone, which could miss functional alterations not associated with gene expression changes. Interestingly, work in the amygdala ^148^ and hippocampus ^164,165^ has also identified astrocyte ensembles and engrams associated with distinct behaviors. Together, these studies significantly extend astrocyte diversity based on molecular differences into the realm of differences based on function and physiology.

### Functions of specific molecules and mechanisms

The previous sections emphasize the roles that astrocytes play in both neuronal physiology and behavioral regulation. Utilizing both gain- and loss-of-function techniques in astrocytes, the findings demonstrate that the dynamics of striatal astrocytes can significantly affect behavior, establishing a causal link between these dynamics and behavioral outcomes. In addition, researchers have made strides in uncovering the molecular mechanisms governing the functions of striatal astrocytes and neurons by investigating the proteins that are common and specific to each cell type.

Proximity-dependent biotinylation methods enabled protein networks found in astrocytes and neurons in mice ^9,166^ to be distinguished. A key finding of the study was the identification of SAPAP3, a protein previously linked to obsessive-compulsive disorder (OCD), which was expressed in both striatal neurons and astrocytes. This protein was found to affect the structure and dynamics of the actin cytoskeleton in astrocytes, leading to reduced astrocyte morphology and territory sizes, which in turn correlated with significant behavioral changes related to OCD. ^9^

Additionally, there has been further exploration of a specific population of astrocytes in the mouse central striatum that express μ-crystallin (*Crym*). ^8,50^ A recent study found that a decrease in μ-crystallin levels in astrocytes of the central striatum led to the development of perseverative behaviors in adult mice. ^117^ This suggests a potential role for *Crym* in modulating certain behaviors, which may correspond to similar mechanisms in humans with compulsive disorders such as OCD and Huntington’s disease (HD), where *Crym* expression is diminished in postmortem brain tissue. ^167,168^ The study found that reduced levels of *Crym* in astrocytes were linked to enhanced synaptic excitation of MSNs. ^117^ The authors propose that this increased excitation results from a loss of astrocytic presynaptic GABAergic control over neurotransmitter release from orbitofrontal cortex projections to the striatum. ^117^ These findings indicate that a disrupted balance between excitatory and inhibitory synaptic transmission plays a role in the behavioral changes observed due to the reduction of μ-crystallin in *Crym*-positive astrocytes. Importantly, by using presynaptic inhibitory chemogenetics, it was found that behavioral and synaptic deficits in *Crym* knockout (KO) mice could be improved, supporting the presynaptic failure of astrocyte control caused by the loss of *Crym*. This suggests that restoring the balance of excitatory and inhibitory responses through targeted interventions ^117^ could provide new treatment options for compulsive behaviors associated with neuropsychiatric disorders. Interestingly, whereas in the mouse *Crym*-positive astrocytes are enriched in the striatum, ^8,50^ in the lamprey they are found in the telencephalon, diencephalon, mesencephalon, and rhombocephalon. ^36^ These features suggest that *Crym-*positive astrocytes emerged early during vertebrate evolution and may have been retained predominantly within the striatum in mice, although evaluations of other mouse brain areas are needed and some *Crym*-positive astrocytes exist in the cortex following neuroinflamation. ^169^ *Crym*-positive striatal astrocytes are observed in RNA-seq studies of humans and marmosets. ^167,170,171^

### Excitatory synapse phagocytosis

A recent study ^172^ investigated the role of astrocytes in synapse reorganization in the striatum, focusing on corticostriatal and thalamostriatal pathways. It highlighted specific MEGF10/MERTK-dependent mechanisms through which astrocytic phagocytosis regulates excitatory synapses, finding that astrocytes preferentially engulf vGlut1-positive corticostriatal synapses while having minimal effects on vGlut2-positive thalamostriatal synapses. This selective phagocytic activity suggests a role for astrocytes in modulating synaptic plasticity and connectivity, which is crucial for motor skill learning. Furthermore, the data suggest that there must exist mechanisms by which astrocytes recognize specific excitatory synapses for phagocytosis. Speaking to the relevance of astrocyte-mediated phagocytosis in the striatum, mice devoid of phagocytic receptors MEGF10/MERTK displayed altered corticostriatal long-term synaptic potentiation and early phase motor learning. Astrocytic phagocytosis of striatal synapses ^172^ thus recalls studies in the hippocampus. ^173,174^

### The shared molecular interface between astrocytes and neurons

The preceding studies illustrate extensive functional and structural interactions between astrocytes and neurons in the striatum. However, the molecular basis of such interactions had not been explored systematically at the protein level for cell surfaces until recently, ^94^ although a study did explore interactions of astrocyte processes with synapses in the cortex with proteomics. ^93^ This was an important topic to tackle because recent large-scale brain cell atlases ^121–125^ have revealed the diversity and coexistence of astrocytes and neurons throughout the CNS in unprecedented detail. High-resolution imaging shows that these cells interact closely within neural circuits, ^8,40,42,81,82,104,175,176^ and evidence from human tissue and mouse models indicates that multiple brain cell types, including neurons and astrocytes, undergo changes in CNS disorders, ^17,52,177–179^ suggesting that brain diseases involve many cell types. In the case of schizophrenia and aging, analysis of postmortem tissue shows that coordinated decreases occur in astrocytic and neuronal synaptic genes. ^180^ However, the specific molecules at the interface between astrocyte and neuron surfaces remained largely unknown, as traditional gene expression studies do not capture these details.

Using extracellularly displayed horseradish peroxidase (HRP) to label cell surface proteins ^181–183^ (CSPs), a recent study mapped the molecular interactions between astrocytes, neurons, and other brain cells. ^94^ The authors found that genes encoding CSPs mediating astrocyte-neuron interactions were dysregulated in several CNS disorders, implicating such multicellular interactions in brain diseases broadly. The major classes of predicted functions mediated by proteins at the cell-surface-shared proteome of astrocytes and neuron (CS SPAN) related to the extracellular matrix, chemical synaptic transmission, cell adhesion, neurotransmitter transport, and regulation of synapse assembly. Molecules involved in these processes include cell-adhesion molecules, transporters, ion channels, GPCRs, synaptogenic cues, and proteins of the extracellular matrix. The study^94^ provides an initial definition of astrocyte and neuron multicellular interactions in proteomic terms and provides a foundation for systematically exploring the astrocyte-neuron interface and its contributions to physiology and brain diseases. However, much more work is needed at the protein level to understand astrocyte-neuron interactions. Candidate mechanisms need to be explored.

## FAILURES OF ASTROCYTE FUNCTIONS AND THE EMERGENCE OF MALFUNCTIONS IN DISEASE

**Figure 6.**
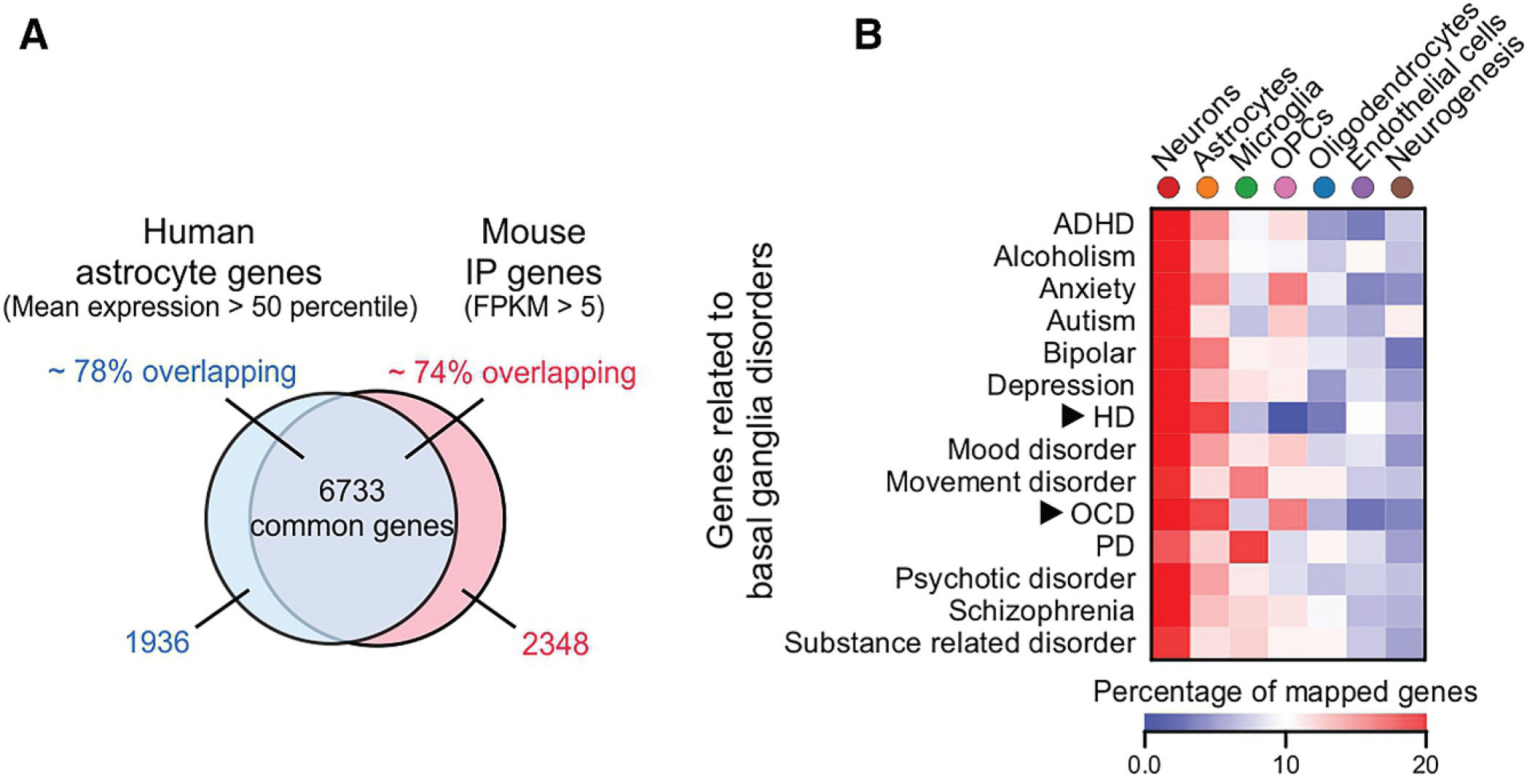
Relative similarity between mouse and human striatal astrocyte gene expression and relationship to disease (A) Venn diagram of overlap between highly expressed astrocyte genes in the human striatum (mean expression > 50th percentile) and mouse striatal IP sample (FPKM > 5) to highlight genes that may be shared. (B) The percentage genes related to BG disorders mapped onto mouse striatal scRNA-seq data based on the top 1,000 cell-type marker genes for the 7 major cell types. Most mapped to neurons. Arrows indicate the two disorders (HD and OCD) with the most astrocyte genes mapped. The figure is adapted from Yu et al. ^18^ Such evaluations are best performed with the latest RNA-seq data for human and mouse tissues available from several cell atlas projects because of the greater numbers of cells and the improved sequencing depth. Progress has been made in exploring striatal astrocyte-neuron interactions for HD and OCD; this work is summarized in this review.

Based on gene expression evaluations, a variety of diseases and disorders could include astrocytes as part of the pathophysiological mechanisms ^184^ –owing to the finding that astrocytes express relevant causative and disease-related genes or because astrocytes display disease-related changes in gene expression, including for the striatum (Figure 6).

### HD

HD is a progressive and severe neurodegenerative disorder marked by motor, cognitive, and psychiatric symptoms. The disease is caused by a polyglutamine expansion in the Huntingtin (HTT) protein, leading to the production of a mutant form (mHTT) that has harmful effects. While HTT and mHTT are found throughout the body, the striatum is particularly vulnerable and exhibits significant atrophy in HD. In both HD mouse models and human HD tissues, striatal astrocytes express mHTT, ^185,186^ and RNA-seq of postmortem HD samples reveals that astrocytes and neurons are significantly altered. Foundational research demonstrated that the expression of mHTT in astrocytes induced slowly emerging HD-related phenotypes in transgenic mice, ^185,187^ while the removal of mHTT from astrocytes in a HD mouse model modestly slowed the progression of some disease symptoms. ^188^ Additionally, the introduction of normal or mHTT-expressing human glial cells into the striatum of HD model and control mice resulted in noticeable improvements and exacerbations of striatum-dependent cellular and behavioral phenomena, ^189,190^ respectively. From an astrocytic perspective, two proteins enriched in astrocytes, Kir4.1 and GLT1, show reduced expression levels in HD mouse models, ^119,191–194^ although whether this occurs in all astrocytes is unclear. These reductions lead to the expected accumulation of K^+^ and glutamate in the extracellular space, partly contributing to the hyperexcitability of MSNs observed in HD models. ^119,120,195,196^ Altered Kir4.1 expression levels have also been shown to be involved in depression-related phenotypes in other brain areas, ^197,198^ and a conditional KO of Kir4.1 is lethal. ^199^

Striatal astrocytes in HD model mice exhibit smaller territory sizes, diminished Ca^2+^ signaling, impaired cholesterol metabolism, and altered GAT function. ^18,120,189^ Furthermore, by identifying astrocyte Gi-GPCRs as upstream regulators of the molecular changes within HD astrocytes, it was found that chemogenetic activation of the Gi-DREADD hM4Di could restore astrocyte morphology, early aspects of behavioral decline in HD model mice, and fast synaptic excitation onto MSNs in a TSP-1 dependent manner. ^18^ Therefore, astrocytic changes are a component of the pathophysiology of HD and progress has been made in dissecting mechanisms. It is, however, worth noting that in the HD mouse models, just like for human disease, HD pathophysiology is unrelenting, and no intervention aimed at astrocytes has restored HD mice to normal. Generally, improvements in early HD-associated phenotypes are observed when astrocyte mechanisms are targeted.

One study suggested the existence of neurotoxic astrocytes in HD based on the expression of the protein C3. ^16^ However, comprehensive analyses of gene expression across multiple mouse models of HD and human postmortem tissue from various stages of the disease ^200^ found that neurotoxic astrocytes were not prevalent.^17^ A study investigating how astrocytes changed in response to HD progression identified a core set of genes and proteins that consistently exhibited altered expression patterns in both animal models and human samples, thereby establishing a potential link between these astrocytic changes and the pathology of HD. ^200^ Many of the altered genes were related to essential functions that astrocytes perform in the brain, including Ca^2+^ signaling, maintaining neurotransmitter balance, and supporting metabolic processes vital for neuronal health. Such alterations suggest that as HD advances, astrocytes progressively lose their capacity to perform these critical normal roles effectively. One significant finding from the study was that by reducing the levels of mHTT specifically in astrocytes using zinc-finger protein transcriptional repressors, ^201^ astrocytes were able to restore the normal expression of many of the identified genes.^200^ This indicates that targeting mHTT levels in astrocytes could represent a viable therapeutic approach to reinstating normal astrocytic support functions and potentially slowing the progression of HD. Moreover, the study emphasized that astrocytes in HD do not merely become reactive, which is a common cellular response to injury and disease. Instead, they begin to lose key functions essential for sustaining neuronal health. This dysfunction can result from intrinsic changes within the astrocytes themselves, known as cell-autonomous changes, as well as from their interactions with other brain cell types, such as neurons. Cells in HD also lose their molecular identity as disease progresses. ^202^

Subsequent research utilizing single-nucleus RNA-seq (snRNA-seq) from individuals with HD has revealed that astrocytes demonstrate state-specific and regionally diverse characteristics during HD pathology. ^171^ These studies identified gene signatures linked to CAG repeat length, lipid-related abnormalities, and highlighted a neuroprotective profile present in a specific astrocytic state that was notably diminished in the caudate nucleus; this depletion correlated with the loss of striatal neurons. The compensatory gene signature in these astrocytes was marked by elevated expression levels of metallothionein genes, and *in vitro* modeling of this state enhanced the viability of neurons derived from HD patients. ^171^ These findings suggest that some astrocytes adopt neuroprotective states in HD. ^171^

Furthermore, a recent study developed a single-cell approach to measure CAG repeat length alongside genome-wide RNA expression in postmortem human tissue. ^202^ This study documented that the HTT CAG repeat can expand somatically from approximately 40 to 45 to between 100 and 500 CAGs in MSNs. Analysis of gene expression revealed that somatic expansion up to 150 CAGs had no significant cell-autonomous effects; however, MSNs with more than 150 CAGs began to lose both positive and negative features of neuronal identity, leading to the de-repression of senescence and apoptosis genes, ultimately resulting in cell loss. Interestingly, astrocytes showed only minor CAG instability, suggesting that degeneration of neurons precedes changes in glial cells. These findings imply that while astrocytes play a role in the pathogenic mechanisms of HD, neurons may be the primary drivers of pathology, ^202^ which had been suggested from experiments employing astrocyte or neuron-selective mHTT reductions. ^203^ Notably, these pioneering studies^202^ identified somatic CAG expansion as a critical mechanism contributing to the loss of MSNs in HD. ^202^ Such a mechanism is prevalent in MSNs.

All the preceding studies suggest that astrocytes and neurons are altered in HD and that both may contribute to pathophysiology. The question of their relative contributions was addressed using ZFP transcriptional repressors to mitigate the expression of mHTT in neurons or astrocytes in mouse models of HD. ^203^ The greatest improvements at molecular, cellular, and behavioral levels were produced by lowering mHTT in neurons, which also significantly improved HD-associated molecular changes in astrocytes. In contrast, mHTT reduction in astrocytes modestly improved HD-associated molecular changes in MSNs. Overall, the key finding was that the primary contributors to pathophysiology in HD are neurons, which exhibit significant dysfunction that accompanies and perhaps drives loss of essential functions in astrocytes. ^203^ Likely mechanisms between astrocytes and neurons mediating such effects include cell-adhesion molecules, and such molecules were disrupted in the shared interface of astrocytes and neurons HD and recovered substantially by mHTT reduction in striatal neurons. ^94^ These data also imply that mHTT reduction in neurons when combined with approaches to restore astrocyte homeostatic functions, such as with endogenous Gi-GPCRs, ^18^ may be useful when exploring new therapeutic strategies.

It is important to note that astrocytic mechanisms could be exploited even though the major effect of mHTT is on neurons. This is because improving astrocytic support of neurons is likely to be beneficial, especially when the neurons are hyperexcitable with greater metabolic requirements. Hence, the fact that mHTT has greater effects in neurons does not rule out important roles for astrocytes in the pathogenesis of HD, e.g., through altered cholesterol metabolism. ^204^ However, it seems unlikely that targeting any single-cell mechanism will fundamentally change the course of HD, which itself is highly complex and involves multiple brain cell types and peripheral organs. ^205^ Instead, targeting astrocytes may represent part of a broader multipronged strategy to address distinct HD phenotypes at different stages of the disease.

### OCD

OCD is a psychiatric disorder characterized by persistent intrusive thoughts (obsessions), repetitive mental or behavioral actions (compulsions), and severe anxiety. ^206^ Obsessions are unwanted thoughts, images, or urges that cause anxiety and distress. Examples include fear of contamination, need for symmetry and order, and disturbing thoughts about harm coming to oneself or others. Compulsions are repetitive behaviors or mental acts that a person feels driven to perform in response to obsessions. Common compulsions include excessive cleaning or hand washing, checking behaviors (e.g., doors and appliances), and counting or repeating words. OCD patients fear negative consequences if a particular compulsion is not performed. Avoidance of compulsion leads to further anxiety. A cycle of events is thus established that includes intrusive thoughts, leading to anxiety and distress that the patient tries to unsuccessfully extinguish by performing the associated compulsion. ^206^

OCD is a chronic, disabling psychiatric condition affecting ∼2%–3% of the population. ^206,207^ OCD is underreported and underdiagnosed, overlapping with related disorders ^206^ such as Tourette syndrome. ^208–214^ This raises important questions about the underlying biology of obsessions, compulsions and repetitive behaviors, that may occur over a continuum. While treatments for OCD exist in the form of cognitive behavioral therapy and medications such as selective serotonin reuptake inhibitors (SSRIs), about half of OCD patients do not respond sufficiently to these first-line treatments. Advances in brain imaging have given rise to important models related to the OCD neurocircuitry^206,215^ and have attributed OCD to arise in a discrete brain circuit composed of the cortex, striatum, and thalamus. This loop, also termed the cortico-striato-thalamo-cortical (CSTC) circuit, ^215^ is altered in OCD patients when compared with unaffected persons, ^216,217^ and it is thought that the key to understanding OCD is to understand molecular, cellular, and circuit mechanisms underlying how the CSTC circuit is altered during compulsive behaviors and OCD. ^28,215^

OCD is polygenic, and genetic studies are much needed to identify risk genes. ^218^ Of the currently known mutations associated with OCD, several map to neurons and glial cells, ^184^ and there is evidence that glia contribute to compulsive phenotypes in model organisms such as worms and mice. ^133,219–222^ Thus, although OCD is a disorder of dysfunctional neural circuits, such studies imply multicellular contributions. ^167^ As with other disorders, it is not feasible to model the full extent of human OCD in mice, ^218^ but there exist useful mouse models ^97^ of compulsive and tic behaviors that are central to OCD and Tourette syndrome. Among these^215,218^ is the deletion of SAPAP3 (*Dlgap3*) that anchors receptors in postsynaptic densities of striatal MSNs ^28,31,206^ and regulates neuronal excitability ^223–226^ and astrocyte morphology. ^9^ SAPAP3 is highly expressed in the striatum and KO mice display anxiety-like phenotypes, tics, and facial lesions due to compulsive self-grooming. ^9,97^ Although unmasking the genetic basis of OCD is an unmet goal, rare missense SAPAP3 variants are observed in OCD, ^227–230^ and *Dlgap3* is downregulated in the OCD striatum. ^167^ Thus, SAPAP3 KO mice provide a useful model to interrogate mechanisms of compulsive behaviors at molecular, cellular, and circuits levels in relation to human studies. It is with the benefit of this model that insights concerning the role of astrocytes in OCD have emerged.

By quantifying the morphology of astrocytes across the CNS and relating this to astrocyte gene expression, ^50^ disease-associated genes for several CNS disorders including OCD were identified. ^50^ Significant enrichment of astrocyte territory related genes was found for OCD, implying that astrocyte morphology changes may occur in OCD. scRNA-seq data had also indicated that striatal astrocytes express genes implicated in OCD ^18^ but did not specifically implicate astrocyte morphology or identify mechanisms. In a separate study, using proteomics, it was found that SAPAP3 was detected at similarly high levels in striatal astrocytes and MSNs^9^; within astrocytes, SAPAP3 was in the cytosol and near the plasma membrane. Overall, multiple approaches provided convergent evidence that astrocytes express SAPAP3, a protein^97^ known to be involved in compulsive behaviors related to OCD.

Striatal astrocyte SAPAP3 interacted with 49 proteins, including those related to the actin cytoskeleton and GPCR signaling, and in accord striatal astrocytes from SAPAP3 KO mice displayed reduced morphology. Conversely, striatal astrocyte-mediated genetic rescue of SAPAP3 corrected compulsive behaviors and astrocyte morphology loss. Furthermore, by identifying Gi-GPCRs as upstream pathways contributing to astrocyte morphology, activation of this pathway with hM4Di DREADDs was found to be sufficient to rescue astrocyte morphology and compulsive behaviors that occurred in SAPAP3 KO mice. In the case of astrocytic SAPAP3 rescue and Gi-GPCR pathway activation, astrocytes improved elevated MSN excitability accompanying ^223–225^ compulsive behaviors in SAPAP3 KO mice. ^9,226^ Together, these studies provide strong evidence that striatal astrocytes contribute to compulsive behaviors in OCD model mice, and the data suggest a pharmacological strategy for ameliorating detrimental disease-related phenotypes in OCD. In addition, there is clear evidence that astrocytic GLT1 is involved in compulsive behaviors related to OCD.^231^ A detailed review on the mechanisms of OCD and how astrocytes contribute has recently been published ^232^ and should be consulted for further details.

### Parkinson’s disease

Astrocytes are known to be become reactive in Parkinson’s disease that includes neuroinflammation, and several genes implicated in Parkinson’s are expressed in both astrocytes and neurons (e.g., *PARK7*, *SNCA*, *PLA2G6*, *ATP13A2*, *LRRK2*, *PINK1*, and *PARK2*). ^233^ Such studies have been reviewed in detail ^233^ and the emerging view is that astrocytes contribute through specific malfunctions related to inflammatory responses, glutamate transport, metabolic dysfunction, cellular stress, and neurotrophic support that Parkinson’s related genes contribute toward. ^233^ In addition, astrocytes will invariably contribute through altered normal functions because of reactive astrogliosis and neuroinflammation, ^17^ in ways specific to Parkinson’s and also shared with other diseases. ^234,235^ Recent studies that explored specific astrocyte mechanisms are discussed below, and the reader is directed to detailed reviews on astrocytes and Parkinson’s disease for additional material. ^233–236^

A recent study explored the impact of GATs, specifically GAT-1 and GAT-3, on DA release within the dorsal striatum, highlighting the role that astrocytes play in this modulation. Although they can work in reverse mode to release GABA, GAT-1, and GAT-3 serve to uptake GABA from the extracellular space, thereby regulating GABA levels within the neuropil. These transporters are found in neurons and astrocytes but are enriched in the DLS relative to the NAc at the protein level. ^162^ The study found that GATs have a crucial effect on the tonic inhibition of DA release. By controlling the ambient concentration of GABA, astrocytes fine-tuned the inhibitory signals arriving onto DA axons, influencing the evoked DA release in the striatum. In the context of a mouse model of early Parkinsonism, the study revealed that a maladaptive downregulation of GAT-1 and GAT-3 occurs, leading to elevated levels of ambient GABA. This increase in GABAergic inhibition results in reduced DA output, which perhaps might contribute to the motor deficits observed in Parkinson’s disease. The findings provide a deeper understanding of the interplay between astrocytes and dopaminergic signaling and underscore the potential of targeting GABA uptake transporters as a therapeutic strategy. By enhancing the activity of these transporters or modulating GABA dynamics, it may be possible to restore normal DA signaling and mitigate some of the symptoms associated with DA dysregulation in neurodegenerative disorders such as Parkinson’s disease. Overall, the study points to astrocytes as active regulators of neuromodulation by DA, which could open new avenues for therapeutic development.

In Parkinson’s disease, long-term treatment with L-Dopa leads to motor complications such as L-Dopa induced dyskinesia (LID). A recent study employing the 6-OHDA mouse model of Parkinson’s with L-Dopa treatment to study the development of LID explored if astrocyte stimulation of Gq and Gi-GPCR pathways was beneficial or detrimental with regard to LID.^237^ The authors found that Gq DREADD activation concomitantly with L-Dopa worsened LID, whereas Gi DREADD activation improved it.^237^ Downstream mechanisms need to be explored and if identified could suggest astrocytic mechanisms that are potentially targetable for LID or for Parkinson’s disease.

### Addiction and drugs of abuse

Since the VS and NAc are critically involved in reward-related behaviors and addiction, the roles of astrocytes are beginning to be explored in detail. A variety of mechanisms have emerged by which astrocytic changes accompany or contribute to behaviors related to addiction and drugs of abuse, including through changes in GFAP expression and astrocyte morphology, ^238–240^ regulation of synaptogenesis, ^156,241^ regulation of the release and uptake of glutamate, ^155,242–244^ release of ATP/adenosine, ^155^ and regulation of astrocyte functional properties. ^245^ The drugs of abuse most often studied are cocaine and opioids, and these studies have recently been extensively reviewed ^44,246–249^ and are not considered further here. Additional studies are much needed to dissect whether astrocytic contributions are largely reactive, regulatory, or causal with respect to distinct phases of behaviors associated with addiction and drugs of abuse. Causative astrocyte roles may be expected in light of elegant experiments that have started to map astrocyte Ca^2+^ responses during stimulation of NAc afferent inputs. ^250^ More recently, a specified subset of astrocytes that emerges during cue-motivated behaviors in the NAc was identified using clever optical and genetic tools, and this ensemble was essential for regulating cue-reward associations. ^143^ Thus, a pharmacological and neural circuit understanding of how astrocytes contribute to reward and addiction-related behaviors is now emerging, portending targeting these cells for therapeutics in substance use disorders.

### Aging

Aging is a major risk factor for neurodegeneration and disease and represents general degradation of functionality with many systems and pathways declining simultaneously. Aging is defined by twelve hallmarks^251^: genomic instability, telomere attrition, epigenetic alterations, loss of proteostasis, disabled macroautophagy, deregulated nutrient-sensing, mitochondrial dysfunction, cellular senescence, stem cell exhaustion, altered intercellular communication, chronic inflammation, and dysbiosis. It seems highly unlikely that astrocytes from any brain region can regulate most of these, but studies of astrocytes could shed light on their contributions to some aging hallmarks, such as neuroinflammation, autophagy, mitochondrial dysfunction, intercellular communication, and senescence in specific brain regions known to contribute to agerelated decline. This is an important topic, because studies in mice and humans demonstrate that aging causes substantial transcriptomic changes to astrocytes and other glia that are more pronounced than those in neurons.^252–255^ Astrocytes are also diverse between brain regions and respond differently to age.^170,256,257^ A detailed study in mice and marmosets used snRNA-seq to show that regional astrocyte identity is patterned before birth and modified postnatally in a way consistent with the view that regional identities are influenced by the needs of local neuronal activity,^170^ as previously suggested.^50^ Substantial numbers of genes were found to be altered within astrocytes with age in both mice and marmosets,^170^ including for the striatum which displayed the highest astrocyte diversity. Critically, species differences with regard to aging-related astrocyte genes were noted between mouse and marmoset,^170^ calling for further studies in relation to human aging.

Using scRNA-seq from young (2 month) and aged (18 month) mice, a recent study (K.E. Linker., V. Duran-Laforet, M. Ollivier, X. Yu, D.P. Schafer, and B.S.K., unpublished data) identified seven molecularly distinct striatal astrocyte clusters. Striatal astrocytes changed significantly with age, exhibiting downregulation of genes, reduced diversity, and a shift to more homogeneous inflammatory transcriptomic profiles. These findings suggest that aging alters regionally enriched striatal astrocytes asymmetrically, with dorsal striatal astrocytes exhibiting greater age-related molecular changes. More generally, recent studies (see K.E. Linker., V. Duran-Laforet, M. Ollivier, X. Yu, D.P. Schafer, and B.S.K., unpublished data; Schroeder et al.^170^ ) lay a solid foundation for cellular and behavioral studies to understand how striatal astrocyte age-related changes contribute to either striatum-dependent behaviors or age-related diseases. It will also be important to determine if age-related changes preferentially affect astrocytes within striatal subregions associated with specific BGs functions that contribute to aging or disease.

### Obesity and metabolism

A recent study ^258^ evaluated striatal astrocyte changes following an obesity-inducing high-fat high-sugar (HFHS) diet in mice and explored the consequences of astrocytic signaling for metabolic and behavioral maladaptive responses following exposure to HFHS. The main findings were that HFHS-induced obesity resulted in astrocyte reactivity in the dorsal striatum and in the NAc. The authors also found that cognitive flexibility was impaired during obesity and improved by hM3Dq chemogenetic activation of striatal astrocytes, which altered striatal neuronal activity. Obesity was found to alter NAc astrocyte Ca^2+^ signaling, and astrocyte hM3Dq chemogenetic activation was shown to change peripheral metabolism and food preferences owing to changes in neuronal function. Taken together these studies ^258^ show that striatal astrocytes are altered during obesity and that their activation can affect whole-body responses. Furthermore, the striatal region-specific changes (e.g., dorsal striatum versus NAc) reported for astrocytes make a strong case that obesity-mediated changes are likely specific to striatal regions. Additional studies to identify how astrocytes regulate striatal neurons in normal lean and in obese mice are warranted and may provide mechanisms concerning the possibility that astrocytes could be exploited to control altered reward-related signaling related to excessive food consumption. ^259^ Since one core function of astrocytes is to provide metabolic support to neurons, it would be interesting to explore how astrocytes are altered in metabolic disorders more broadly and how such changes contribute to their CNS phenotypic manifestations.

### Astrocyte-neuron conversion

In mice, local damage such as stroke results in suppression of Notch signaling and recruits striatal astrocytes outside known neurogenic niches into a neurogenic program that is proposed to generate striatal neurons. ^259^ Subsequent studies showed that such neurons are unlike endogenous striatal neurons and are glutamatergic. ^260^ These studies raise the possibility of reprogramming striatal astrocytes into neurons in the context of neurodegeneration and injury. Several studies have also reported conversion of striatal astrocytes to neurons with single or combinations of transcription factors, knockdown of a RNA splicing factor, or chemically (e.g., DLX2, NeuroD1, ASCL1, LMX1A, and Ptbp1) ^261,262^ –including in models of HD ^263^ and Parkinson’s. ^264^ However, the degree to which these procedures can generate mature functioning neurons that project to appropriate targets and integrate into functioning neural circuits in meaningful and beneficial ways is not yet understood. The topic of astrocyte-neuron conversion is rapidly developing with data both supporting and challenging astrocyte-neuron conversion as a phenomenon. A comprehensive assessment of this work is beyond the scope of this review focused on astrocyte-neuron interactions. Issues relevant to the possibility and potential of astrocyte-neuron conversion have been discussed, ^265–267^ including in a useful commentary. ^268^ Key technical issues relate to the specificity of astrocyte labeling and the adequacy of lineage tracing methods. In addition, it may be worth reconsidering the logic of disrupting naturally balanced neuron-astrocyte ratios and interactions as discussed in this review by converting the CNS’s major support cells to neurons at the expense of their normal essential roles in tissue homeostasis that may be especially important in disease settings.

## OUTLOOK AND FUTURE DIRECTIONS

1. *At a molecular level*, it will be critical to explore the functions of astrocyte subsets for physiology. Such experiments will require the development of tools to target specific subsets. The use of enhancer AAV constructs has provided tools to target neuronal subsets, ^269^ and it may be possible to do so for astrocytes. ^270^ Once such tools are available, entirely new questions will emerge at the microcircuit level and how astrocytes are integrated locally within subregions of brain areas. Astrocyte diversity could thus be explored at the functional level. Since astrocytes are morphologically complex, it seems feasible that subcompartment specific proteins exist and would furnish the tools needed to manipulate such subcompartments selectively and determine functional consequences. Impressive volume electron microscopy^40^ has revealed the complexity of astrocytes at nanometer resolution, and such studies need to be expanded across the brain to determine how regional specialization is reflected in astrocyte structure. The molecular basis of astrocyte tiling and morphology both need to be explored. ^80^ The astrocyte-neuron interface is replete with cell-adhesion and extracellular matrix molecules, ^94^ but the functions of most from an astrocytic perspective are unexplored. The functions of the many core genes shared across astrocytes from different CNS regions need to be fully understood, including those involved in tissue homeostasis and metabolism.
2. *At the cellular level* of astrocyte Ca^2+^ signaling, we need biophysical and predictive models of astrocyte Ca^2+^ dynamics in cells with realistic shapes and we need a molecular understanding of how astrocyte Ca^2+^ signals are coordinated, which will be greatly aided by recently improved analysis methods. ^271^ A recent proposal for astrocytes as being involved in contextual guidance ^27^ has great merit and needs to be tested. A proposal ^98^ based on studies in *Drosophila* and forebrain astrocyte cultures that pre-exposure to noradrenaline gates subsequent astrocyte responsiveness to neuromodulators such as DA similarly deserves to be fully evaluated for vertebrates. The suggestion that noradrenaline gates ^98^ responses to common other neuromodulators is challenged by studies over ∼30 years that reported neuromodulator-evoked Ca^2+^ responses without pre-exposure to noradrenaline (see Fiacco et al.,^24^ Semyanov et al.,^64^ Khakh and McCarthy, ^135^ Verkhratsky and Nedergaard, ^272^ Volterra et al., ^273^ Lines et al.,^274^ Bazargani and Attwell, ^275^ and Porter and McCarthy ^276^ for references). However, perhaps in accord with the gating idea in the case of the striatum, NAc astrocytes responded to DA robustly with Ca^2+^ elevations, ^155^ but those in the dorsal striatum responded only weakly.^8^ It therefore seems possible that the proposed gating varies between brain regions. Astrocytes also release ATP and respond to it robustly via ATP receptors with Ca^2+^ elevations. ^277–279^ There is no report that such experiments required pre-exposure of astrocytes to noradrenaline for such signaling to occur, implying that the gating idea is not applicable to all neuromodulators and GPCRs such as ATP receptors. ^280^ However, the gating idea is interesting, potentially important physiologically, and needs to be tested carefully in vertebrates with brain slice and *in vivo* studies.
  In relation to Ca^2+^ signaling more generally, GPCR responses can be strongly influenced by the type of receptor agonist employed (e.g., full or partial agonists). ^281^ Thus, the Black/Leff operational model establishes that GPCR sensitivity to partial agonists can be altered by changes in tissue sensitivity, ^282,283^ which could be altered by prior agonist responses. Agonist concentration-effect curves ^284^ can be shifted rightward following receptor desensitization, which may be expected to occur when only low doses of drugs are tested after a higher dose. Furthermore, since astrocytes display multiple forms of Ca^2+^ dynamics in different compartments that can be studied reliably with semiautomated data analysis, ^271,285–287^ it will be important to study GPCR responses accordingly rather than through aggregate analysis of whole fields of view that likely bias somatic responses. Given that astrocytes express many GPCRs, these considerations call for care and detailed pharmacological studies. Some of these topics have been discussed already at some length in reviews and book chapters. ^5,64,135,136^
3. *At the level of circuits and behavior*, the availability of tools, molecular mechanisms, and appropriate analysis of Ca^2+^ signals will permit the parsing out of how astrocytes regulate circuits to alter behavior. As a field, we need to move away from simply saying neurons fire differently and begin to understand the logic of how and why astrocytic regulation of circuits and behavior is important. Contextual guidance is a useful framework to begin to perform these experiments, ^27^ and such studies will need to be combined with computational approaches. ^288^ Theoretical approaches such as those that are emerging for astrocyte-neuron interactions ^289,290^ are needed to inform new experiments. Pioneering recent studies have identified astrocyte ensembles ^143^ and engrams ^148,164^ linked to specific behaviors such as cue-reward associations and memory, as well as astrocytes that display immune-related memory. ^291^ Such mechanisms need to be explored for different circuits, brain regions, and behaviors. The precise functions served by the complex morphology of astrocytes also need to be delineated. Studies show that astrocytes possess primary cilia, but that they are absent from other glia (microglia and oligodendrocytes). ^292^ It will be important to explore astrocytic primary cilia functions within the context of neural circuits.
4. *At a disease level*, the striatum is relevant for investigating the relationship between astrocytic signaling and neuropsychiatric behaviors and neurological disease in mouse models. Moreover, careful analysis of changes in striatal astrocyte gene expression following experimental manipulations indicated that astrocytes respond in highly context-dependent ways, ^18^ which was confirmed across CNS disorders. ^19^ This suggests that mechanisms related to specific diseases and behaviors may exist within astrocytes, offering opportunities for further exploration and potential therapeutic exploitation. The relationship to HD, OCD, PD, and addiction means the striatum could be in the forefront of studies that begin to exploit astrocytes for translational work. It will be important to rigorously determine if astrocytes are amenable to pharmacological or genetic manipulation to produce desirable effects in the circuits that drive complex behaviors related to disease. In the case of depression ^197,198^ and compulsive behaviors, ^18,225^ there is good evidence that this is the case. If so, astrocytes may be exploitable to develop novel therapeutics for human disorders. Mechanisms within astrocytes that are parallel and orthogonal to the progression of the underlying disease mechanism could be exploited. We have not considered reactive astrogliosis in this review (recent reviews are useful, ^17,293,294^ however). Nonetheless, the functional effects of reactive astrogliosis on neurons and circuits have not been satisfactorily dissected and this requires detailed work. How astrocytes from different regions change with aging also needs to be explored at different biological scales. Furthermore, it is probable that astrocyte malfunction precedes overt reactive astrogliosis and targeting such earlier mechanisms may be tractable. We need better *in vitro* models of human astrocytes that reflect age- and disease-related changes that accumulate over life.

Astrocytes are fascinating complex cells (Figure 1), and once seen under the microscope, it is impossible to unsee them. The next few years promise to be exciting for those interested in the fundamental biology of astrocyte-neuron interactions, multicellular CNS physiology, and in advancing novel therapies for disease. One expects astrocytes will reveal more of their secrets as the field grows and continues to attract new researchers. For astrocyte-neuron interactions, key goals will be molecular and mechanistic understanding^37^ of astrocytes and to translate glial biology discoveries into new treatments and diagnostics for human disease. Such studies require model organisms and human material to arrive at mechanisms that can be exploited. Research on astrocyte-neuron interactions is thus aligned with the priorities of national, international, and philanthropic research agencies, and importantly with the expectations of modern society to deliver on the promise of basic research.

## Box 1. Definitions

### Neural microcircuit:

In the case of circuits, we adopt Sten Grillner’s anatomical definition ^38^ of a neural microcircuit as “comprising neurons and associated cells such as glia, organized to carry out specific operations within a region of the nervous system.”

### Astrocyte processes at synapses:

The term “tripartite synapse ^39^” was coined to convey astrocytic processes as the third element of synapses with pre- and postsynaptic elements. Subsequent work has shown that not all synapses contain astrocytic contacts, and those that do often display variable coverage of pre- and/or postsynaptic elements. ^40–42^ Furthermore, some synapses have microglial processes, ^43^ and perhaps other cellular elements that cannot be captured with current optical techniques. Thus, the term tripartite synapse, although useful as an early concept, has lost its meaning and has recently been revised to describe synapses more accurately as multipartite ^44^ comprising processes of diverse cells and the extracellular matrix. Sherrington’s original term “synapse” from the Greek meaning “to clasp” based on meticulous anatomical and physiological studies where a specialized area separates connected nerve cells, ^45^ however, seems adequate for our purposes. Indeed, Sherrington was prophetic when he outlined possible functions of the surface between synaptic elements.

Such a surface might restrain diffusion, bank up osmotic pressure, restrict the movement of ions, accumulate electric charges, support a double electric layer, alter in shape and surface tension with changes in difference of potential... or intervene as a membrane between dilute solutions of electrolytes of different concentration or colloidal suspensions with different sign of charge.

Several of these possible functions, almost completely unstudied at the time of Sherrington, could now be correctly ascribed at least partly to astrocyte processes. We use Sherrington’s definition of the synapse because it seems sufficiently accurate and is widely accepted in neuroscience.

### Cell-cell signaling:

Intercellular signaling mediated by diffusible messengers, electrical junctions, ephaptic coupling or direct contact-mediated mechanisms is a universal feature of multicellular life. In this review, cell-cell signaling is used as the framework to interpret how astrocytes interact with neurons. The term “gliotransmission” that is often used to convey neurotransmission-like phenomena from astrocytes to neurons is not used because it does not fully capture the multiplicity of ways in which astrocytes regulate neurons and because it remains controversial. ^23,46^

### Neuroimmune circuit:

In the case of neuroimmune circuits, they comprise complex wired and signaling communication networks that connect the nervous system and immune system, allowing bidirectional interactions between these major physiological systems. ^47^ Astrocyte responses during neuroimmune interactions are not considered in detail because this topic is extensively reviewed. ^47,48^
